# Canola grain yield and quality response to Sunn hemp cover crop, combined nano zinc and copper, and nitrogen fertiliser application under different agroecological zones

**DOI:** 10.3389/fpls.2025.1706625

**Published:** 2025-12-01

**Authors:** Mahlare Mapula Mokgophi, Kingsley Kwabena Ayisi, Pholosho Mmateko Kgopa, Mapotso Anna Kena

**Affiliations:** 1 Centre for Global Change, University of Limpopo, Sovenga, South Africa; 2 Department of Plant Production, Soil Science and Agricultural Engineering, University of Limpopo, Sovenga, South Africa

**Keywords:** canola production, oil yield, cover crop, nanofertiliser, protein yield, agroecological zones

## Abstract

**Introduction:**

Canola production requires considerable nitrogen fertilisation, which negatively impacts the environment. This study evaluated the effects of Sunn hemp cover crop, nano zinc and copper, and nitrogen fertiliser on grain, oil and protein yields at Syferkuil and Ofcolaco during 2023 and 2024.

**Methods:**

Cover crop was planted, slashed, and incorporated into the soil 60 days after planting, then followed by canola in a split split-plot design with 16 treatments and 3 replications. Statistical analysis was conducted using JASP software 0.19.3.

**Results:**

Results showed increases in grain yield (17% to 43%), oil yield (21% to 68%), and protein yield (21% to 47%) with cover crop incorporation, although location-specific variances were noted. At Syferkuil, nano zinc and copper enhanced grain yield by 15% to 17% and protein yield by 16% to 27%. Nitrogen fertilisation at 120 kgN ha-1 also resulted in higher oil yields, which was comparable to 180 kgN ha-1, although this varied across seasons and locations.

**Discussion:**

Overall, cover crop, nano zinc and copper can reduce nitrogen inputs while maintaining oil yield depending on specific conditions.

## Introduction

*Brassica napus L.* (Canola) is an important crop globally cultivated for its seeds, which are a rich source of vegetable oil containing an average of 43% as well as protein constituting an average of 19% of its total composition (Poisson et al., 2019). The crop is a valuable source of oilseed in several countries due to its high-quality oil, which is rich in omega-3 fatty acids and vitamin E, and contains minimal saturated fatty acids, among other attributes. Canola is occasionally planted after legume crops to utilise the residual mineral N content in the soil (Kirkegaard et al., 2016). The substantial nitrogen demands of the crop render optimal fertiliser management challenging, particularly in regions experiencing variable rainfall.

Chemical fertilisers, being the main source of N in canola production, play a pivotal role in increasing crop yield due to their role in the plant metabolism system, where all essential plant-related processes are strongly correlated with N (Gomaa and Fayed, 2024). The crop has low nitrogen use efficiency which negatively affects photosynthetic processes as well as leaf growth parameters, subsequently resulting in decreased yield (Zhuo et al., 2024). Essentially, the appropriate N fertilisation regime can enhance the vegetative and reproductive development of canola, potentially resulting in yield and biomass increases (Lin et al., 2020). Fertilisation beyond the crop’s demand has detrimental effects on the soil quality and overall environment (Eibad et al., 2022).

Incorporating cover crops such as sunn hemp (*Crotalaria juncea*) promotes sustainable agricultural practices in cropping systems through improved microbial activity, nutrient cycling, and distribution (Koudahe et al., 2022). Cover crops can mitigate the effects of climate change through biological N fixation and carbon sequestration, thus curbing their losses to the atmosphere (Blanco-Canqui et al., 2013a). Additionally, numerous research studies have elucidated the benefits of nanofertilisers on crop yields and soil productivity due to their nanoscale (Babu et al., 2022; Hu and Xianyu, 2021). Micronutrients such as Zn and Cu are essential in crop production given their roles in chlorophyll production, respiration, resistance to plant diseases, and the activation of enzymes in plant systems that are responsible for electron transport among others (Lohry, 2007). Global adoption of nanofertilisers could be pivotal in achieving sustainable agriculture because of their high bioavailability (Irewale et al., 2024) and slow-release mechanisms, among other characteristics (Mahapatra et al., 2022). The novelty of this study lies in establishing that optimum canola oil yield can be achieved through the combined application of cover crop and nano Zn and Cu, allowing for the use of less N fertiliser, thus addressing the environmental effects of excessive fertilisation. Hence, the study was aimed to assess the combined effect of sunn hemp cover crop, nano Zn and Cu, and N fertiliser on canola grain yield and quality.

## Materials and methods

### Study site description

**Figure 1:**
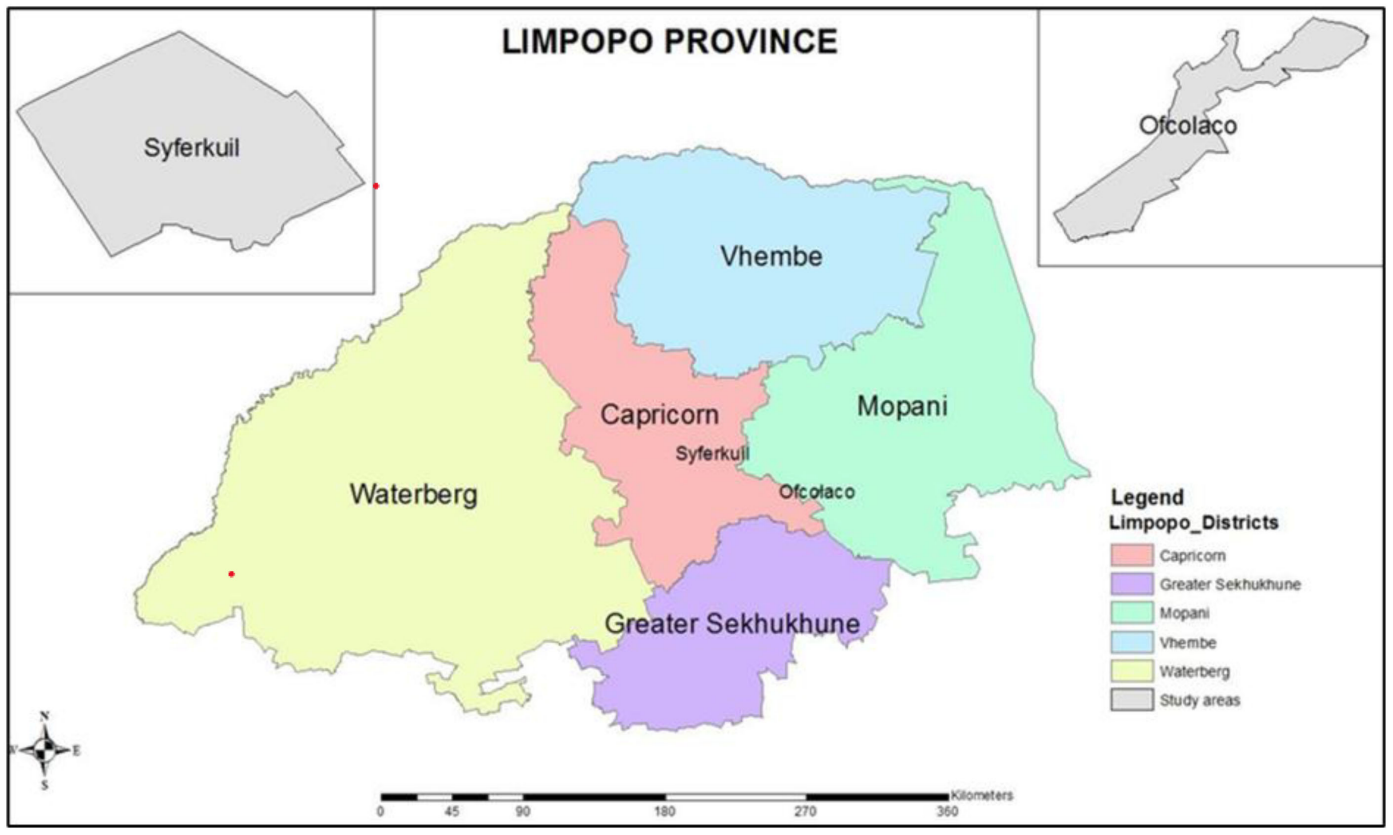
Study location map for Syferkuil and Ofcolaco in Limpopo province (reproduced from Masete et al., 2022, licensed CC-BY-4.0).

The study was conducted at two agro-ecologically diverse locations in the Limpopo province of South Africa, namely, the University of Limpopo experimental farm (Syferkuil) and a Cooperative Farmers’ field at Ofcolaco in Capricorn and Mopani Districts, respectively, in the 2023 and 2024 growing seasons (**Figure 1**). The Syferkuil farm is in the Polokwane Local Municipality at geographical coordinates of 23°51′0″S, 29°42′0″E. The Ofcolaco farmers’ field is in the Maruleng Local Municipality, approximately 120 km south-east of Syferkuil at coordinates of 23˚56′S,31˚07′E. Syferkuil receives an average annual rainfall of 450 mm. In contrast, the semi-arid zone of Ofcolaco, located in the Maruleng municipality, receives approximately 700 mm of annual rainfall, predominantly during the summer season. Both areas are dominated by Hutton soil form (Soil Classification Working Group, 1991), with Syferkuil topsoil containing 26% clay and Ofcolaco having 32% clay.

### Weather conditions for the growing season

**Figure 2:**
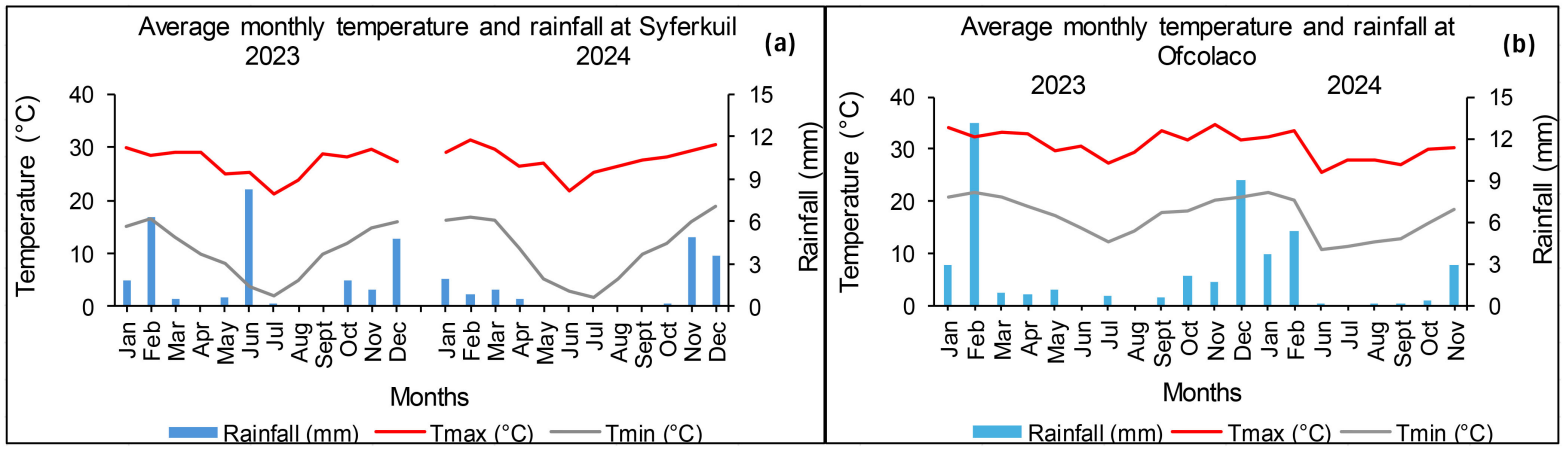
Average monthly rainfall and maximum and minimum temperatures at Syferkuil **(a)** and Ofcolaco **(b)** for 2023 and 2024.

The monthly maximum temperatures at Syferkuil ranged from 21.28 °C to 29.83 °C in 2023, whereas in 2024, they ranged from 28.93 °C to 30.49 °C. On the other hand, minimum temperatures ranged from 1.85 °C to 16.67 °C in 2023 and 18.18 °C to 18.74 °C in 2024. For the duration of the growing season, the location received 25.71 and 13.25 mm average rainfall in 2023 and 2024, respectively (**Figure 2a**). On the other hand, Ofcolaco had monthly temperatures with a maximum range of 27.23 °C to 34.78 °C and a minimum of 12.22 °C to 21.65 °C. The average seasonal rainfall was 32.92 and 12.42 mm for the duration of the 2023 and 2024 seasons, respectively, with the highest rainfall received in February (**Figure 2b**).

### Site management and experimental layout

The fields at Syferkuil and Ofcolaco were each demarcated into equal portions, with one-half used for cover cropping, whereas the other remained fallow for the duration of cover crop growth. Prior to planting canola in winter, sunn hemp was planted on the experimental units assigned to the cover crop in summer (December to February for 2022 and 2023), slashed at the onset of flowering, and left *in situ* at Syferkuil and Ofcolaco. The covered and non-covered plots were disked and allowed a resting period of 4 weeks before the canola was planted. The canola trial was established in winter (April to September for 2023 and 2024) as a split split-plot design with 16 treatments and three replications (blocks).

**Figure 3:**
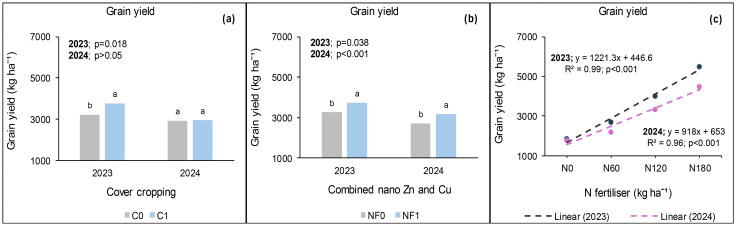
Effects of cover crop **(a)**, combined nano Zn and Cu **(b)** and N fertiliser **(c)** on grain yield at Syferkuil. C_0_: without cover crop; C_1_: with cover crop; NF_0_: no combined nano Zn and Cu; NF_1_: with combined nano Zn and Cu.

The main plot treatment was assigned to cover crop (with (C_1_) and without (C_0_)), the subplot was combined with nano Zn and Cu micronutrient (with (NF_1_) and without (NF_0_)), and the sub-subplot was applied N fertiliser rates (0, 60, 120, and 180 kg N ha^−1^) labelled as N_0_, N_60_, N_120_, and N_180_, respectively (**Figure 3**). The combined nano Zn and Cu micronutrient fertiliser is a clear blue solution containing Cupric Nitrate 3H_2_O (54.8 g L^−1^), Zinc Nitrate 6H_2_O (262 g L^−1^), and Silver Nitrate (1.66 g L^−1^). The particle size of the nanofertiliser ranges between 1 and 100 nm and has an undiluted pH of <1.5. This was applied as a foliar spray once weekly for 4 weeks after emergence and then biweekly until flowering with a dose of 20 mL/100 L of water using a knapsack with a flat fan nozzle. Canola was planted within experimental units measuring 3 m × 4 m, with 90-cm inter-row spacing and 15-cm intra-row spacing, with buffering spaces of 1 and 1.5 m between plots and replications. Nitrogen fertiliser was applied as Lime ammonium nitrate (LAN) (28% N) at Ofcolaco and Urea (46-0-0) at Syferkuil in a split application, half at planting and the other half at flowering. The crops were irrigated using a sprinkler system at both locations. For sun hemp cover crop, the crops were irrigated during the initial stages of growth until full crop establishment and then continued as dryland until incorporation into the soil. Canola plants were irrigated at 30 mL per week, measured by rain gauges from seeding to physiological maturity. Both sites used similar land preparation and weeding techniques, which were disking and chemical weed control, and previously used the herbicide Roundup (glyphosate N-(phosphonomethyl) glycine) and hand hoeing.

### Soil sampling and analysis

Eight soil samples were collected randomly from each study area at a depth of 0–30 cm and composited into two for pre-plant soil analysis. The soil samples were air-dried and sieved through a 2-mm mesh and stored in a sterile zip-lock plastic bag for chemical property analysis.

Total N was measured using a block digester (SC154 Hot Block Digester, Environmental Express, Charleston, USA) following the Kjeldahl method by Bremner (1996), where 1 g of soil sample was transferred into a 100-mL digestion tube. A catalyst mixture of 1–2 g was added, followed by 3–5 mL of concentrated sulphuric acid (in fume hood), and the contents were swirled until thoroughly mixed. Digestion tubes were placed in the block digester at 390°C, ensuring not to exceed 410°C. The mixture was boiled until clear and then removed from the digester to cool. About 20 mL water was added to each tube, and then 20 mL H_3_BO_3_ was also added and distilled at 22°C. A few drops of indicator (0.10 g Bromocresol green and 0.07 g methyl red in 100 mL ethanol (96%)) were added, and the solution was titrated with 0.01 mol L^−1^ sulphuric acid. The solution was then read on a gallery analyser.

Available phosphorus (P) was extracted using a 1% citric acid extraction method by Blanck (1931). A soil sample of 5 g was used, where 50 mL of 1% citric acid solution (dissolved 11 g citric acid in distilled water to make 1 L) was added to the sample and placed on a reciprocating shaker for 2 h. This mixture was allowed to stand for 20 h and then placed on the shaker for an additional hour and then filtered. After filtering, 1 mL of the filtrate was poured into a 100-mL volumetric flask, and 80 mL of distilled water and 10 mL of mixed reagent (dissolved 1.5 g ascorbic acid in 100 mL stock solution) were added. This solution was allowed to stand for an hour, and then absorbance was read on a UV/VIS spectrophotometer (Thermo Fisher Scientific, Helide Gamma, New York, USA) at a wavelength of 882 or 720 nm.

The extractable cations (K, Ca, and Mg) were measured using 1 M NH_4_OAc (Okalebo et al., 2002), whereas micronutrients (Cu and Zn) were extracted using 0.25 M ethylene diamine tetra-acetic acid (EDTA) (Garcia et al., 1993) and read on an ICP-9000 emission spectrometer.

Soil pH was extracted by adding 50 mL deionised water to 10 g soil and stirring the mixture for 10 min and then allowed to stand for 30 min, followed by a 2-min stirring, after which pH in the suspension was measured using a pH meter (HI2002–01 edge, Hanna instruments, UK) following the electrode method (Thomas, 1996).

Organic carbon (OC) was determined by following the Walkley–Black chromic acid wet oxidation method (Walkley and Black, 1934). A 10-mL K_2_Cr_2_O_7_ solution was added to 1 g of sample, and then 20 mL sulphuric acid was added and allowed to cool for 30 min. After cooling, 150 mL of de-ionised water and 10 mL concentrated orthophosphoric acid were added, and the excess K_2_Cr_2_O_7_ was titrated with iron(II) ammonium sulphate after adding 1 mL of indicator (barium diphenylamine sulphonate). Organic carbon content was then calculated using the following formula:


$$[\text{Fe}{\left({\text{NH}}_{4}\right)}_{2}{\left({\text{SO}}_{4}\right)}_{2}{\text{ moldm}}^{ˉ3}]=\frac{\left[10{\text{cm}}^{3}{\text{ K}}_{2}{\text{Cr}}_{2}{\text{O}}_{7\;}\times 0.167\times 6\right]}{\left[{\text{cm}}^{3}\text{ Fe }{\left({\text{NH}}_{4}\right)}_{2}{\left({\text{SO}}_{4}\right)}_{2}\right]}$$

$$\%\;OC=\frac{\left[{\text{cm}}^{3}\text{Fe}{\left({\text{NH}}_{4}\right)}_{2}\text{ blank}ˉ{\text{cm}}^{3}\text{Fe}{\left({\text{NH}}_{4}\right)}_{2}\text{ sample]}\times [\text{M x }0.3\;\times \text{ f}\right]}{\text{g sample}}$$

where *M* = concentration of Fe (NH_4_)_2_(SO_4_)_2_ and f = 1.3.

### Pre-sown soil properties of the experimental field

The pre-sown soil test results showed that Syferkuil had alkaline pH, whereas Ofcolaco was slightly alkaline post sunn hemp incorporation. Sufficient to slightly high concentrations of P, K, Mg, Cu, and Zn were observed at both locations as per the critical soil test limits, before and after sunn hemp incorporation. After sunn hemp incorporation, Syferkuil improved in Mg, P, Zn, total N, and organic carbon. Ofcolaco showed similar trends in nutrient improvements except Ca, Mg, K, P, and Cu (**Table 1**).

**Table 1:** Pre-plant soil physicochemical properties at the two experimental sites.

Soil properties	Syferkuil			Ofcolaco			Soil limits
	Before sunn hemp	After sunn hemp	Difference	Before sunn hemp	After sunn hemp	Difference	
pH_(H_2_O)_	8.88	8.70	−	7.91	6.78	−	-
^¥^Ca	846	799	−	1208	728	−	>2,000
^¥^Mg	619	629	+	394	163	−	84-600
^¥^K	316	261	−	220	143	−	80-200
^¥^P	64	69	+	117	104	−	>15
^¥^Cu	4.61	3.73	−	7.41	5.19	−	0.75-100
^¥^Zn	5.98	6.76	+	7.43	8.88	+	3-150
N (%)	0.08	0.09	+	0.06	0.07	+	-
OC (%)	0.77	0.85	+	0.75	1.15	+	-
Texture	Sandy clay loam			Clay loam			

^*Critical soil test limits adapted from:^Fertiliser Society of South Africa (FSSA-MVSA) (2007); Landon (1991); Havlin et al. (2013), and Horneck et al. (2011).

¥ nutrients in (mg kg^−1^). Aggregate stability: MWD in (mm).

## Data collection

### Seed yield and yield components

Seed yield was obtained from a 2-m^2^ area in the centre of each plot, excluding the boundaries at both locations, and the harvested pods were subsequently threshed and weighed. The number of seeds per pod was counted from an average of 10 pods, whereas the number of pods per plant was counted from an average of five plants. Thousand-seed weight was determined by manually counting the seeds and then weighing them.

### Oil yield

Seed oil and protein were analysed following the method of A.O.A.C (1990). The seed oil and protein yield were calculated as follows:


$$Oil\;yield\;(kg\;ha^{-1})=seed\;oil\;(\%)\;*\;grain\;yield\;(kg\;ha^{-1})$$

$$Protein\;yield\;(kg\;ha^{-1})=nitrogen\;(\%)\;*\;6.25\;*\;grain\;yield\;(kg\;ha^{-1})$$

The harvest index was calculated as follows:


$$HI\;(\%)=\frac{grain\;yield\;(kg\;ha{ˉ}^{1})}{grain\;yield\;(kg\;ha{ˉ}^{1})+biological\;yield(kg\;ha{ˉ}^{1})}$$

### Statistical analysis

A standard analyses of variance (ANOVA) was conducted using Jeffrey’s Amazing Statistical Program (JASP 0.19.3) to detect treatment effects and interactions for the selected yield parameters. Tukey’s Honestly Significant Difference (HSD) test was also used to compare treatment means at a probability level p<0.05. Normality and homogeneity of variance tests were conducted through skewness and kurtosis, as well as Levene’s test for equality of variance. Regression analysis was carried out to compare treatments and establish the extent of relationships among the measured parameters. Principal component analysis was performed, where rotated components (RC’s) with eigenvalues above 1 were retained.

## Results

### Grain yield at Syferkuil

During the 2023 cropping season, cover crop, combined nano Zn and Cu, and N fertiliser significantly affected grain yield, but their interaction was not significant. Cover crop incorporation increased grain yield by 17% compared with no cover crop (**Figure 3a**). Meanwhile, combined nano Zn and Cu increased grain yield by 15% relative to no application (**Figure 3b**). Nitrogen fertilisation at 180 kg N ha^−1^ increased grain yield by 198% and 37%, respectively, compared with 0 and 120 kg N ha^−1^ (**Figure 3c**). Furthermore, under 120 kg N ha^−1^, grain yield was 72% that of 180 kg N ha^−1^.

**Figure 4:**
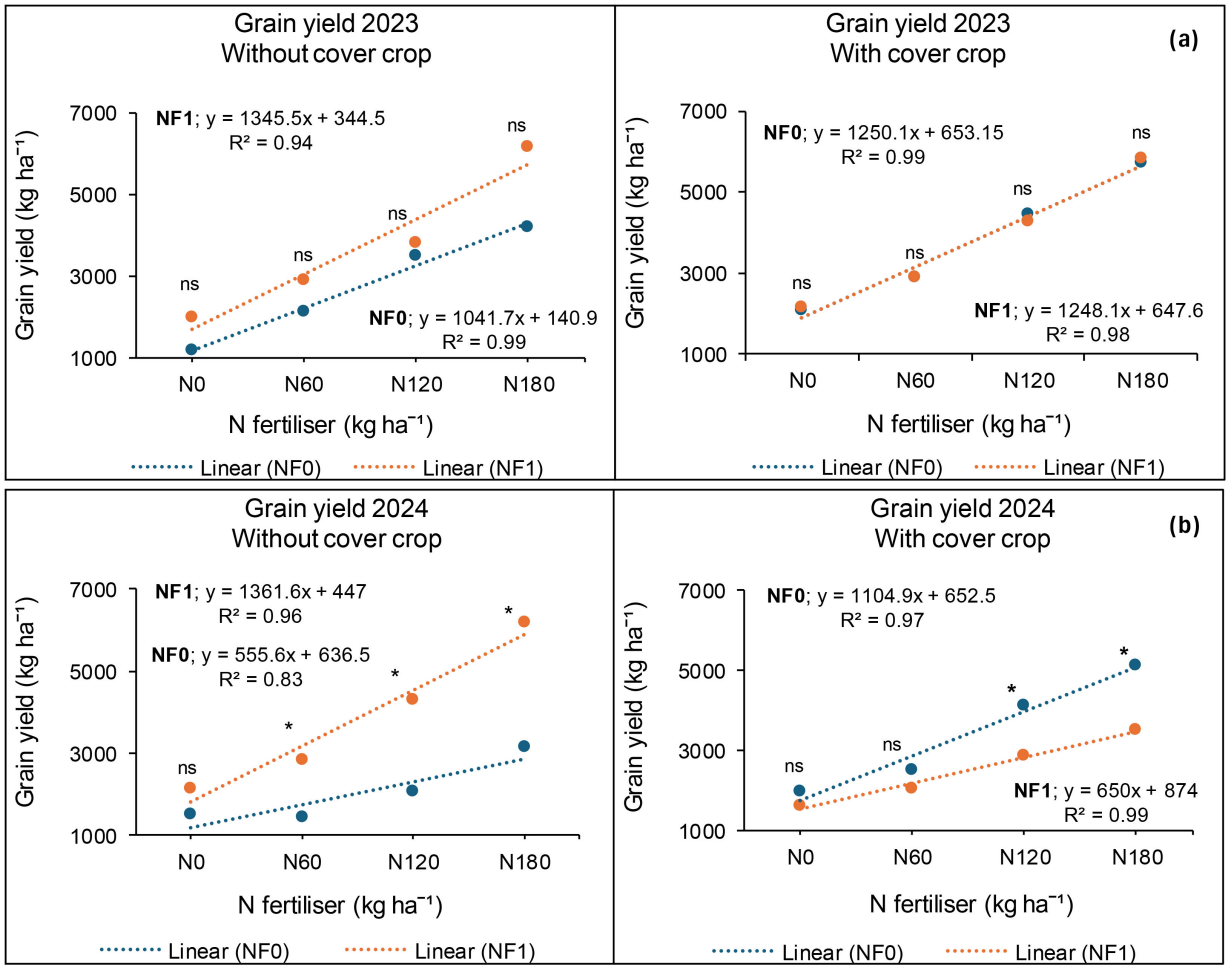
Cover crop, combined nano Zn and Cu, and N fertiliser on grain yield at Syferkuil for 2023 **(a)** and 2024 **(b)**. C_0_: without cover crop; C_1_: with cover crop; Nf_0_: no combined nano Zn and Cu; NF_1_: with combined nano Zn and Cu.

In 2024, grain yield was significantly affected by combined nano Zn and Cu and N fertiliser, but not by cover crop. The interactive effect was only observed between cover crop, combined nano Zn and Cu, and N fertiliser. The application of nano Zn and Cu resulted in 17% more seed yield than the plots which had none (**Figure 3b**). Furthermore, N fertiliser resulted in a 149% increase under 180 kg N ha^−1^ relative to zero application and 62% compared with the average of 60 and 120 kg N ha^−1^ (**Figure 3c**). Additionally, without the application of cover crop, and nano Zn and Cu, canola grain yield obtained under 120 kg N ha^−1^ was comparable with that of 180 kg N ha^−1^ (**Figure 4b**). On the other hand, grain yield was 114% higher under 180 kg N ha^−1^ compared with the average of 0 and 60 kg N ha^−1^ (**Figure 4b**). Furthermore, from the grain yield obtained under 180 kg N ha^−1^, 120 kg N ha^−1^ constituted 69% when combined nano Zn and Cu was applied without a cover crop (**Figure 4b**). Under cover crop incorporation, N fertilisation at 180 kg N ha^−1^ increased grain yield by 129% and 44%, respectively, compared with the average of 0 and 60 kg N ha^−1^; and 120 kg N ha^−1^ without nano Zn and Cu application (**Figure 4b**). Lastly, combining cover crop and nano Zn and Cu with N fertiliser at 180 kg N ha^−1^ increased grain yield by 92% compared with the average of 0 and 60 kg N ha^−1^ (**Figure 4b**).

### Grain yield at Ofcolaco

**Figure 5:**
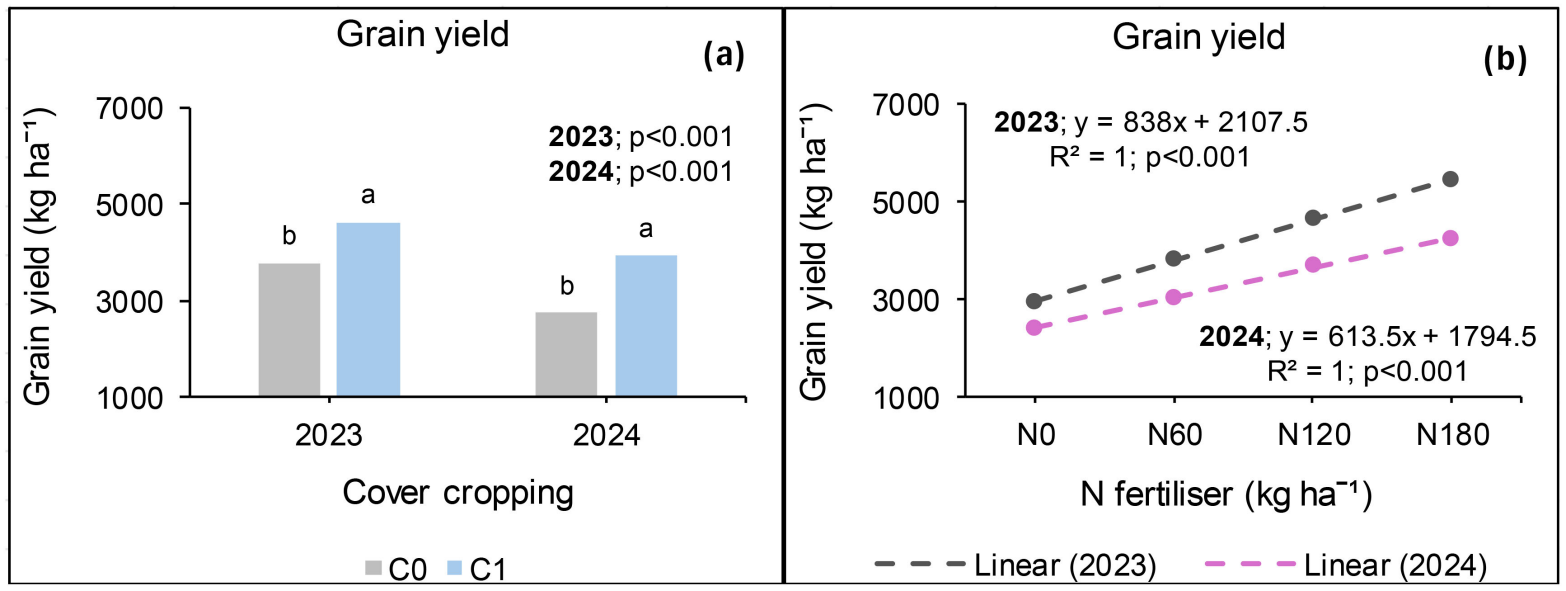
Effects of cover crop **(a)** and N fertiliser **(b)** on grain yield in 2023 and 2024 at Ofcolaco. C_0_: without cover crop; C_1_: with cover crop.

**Figure 6:**
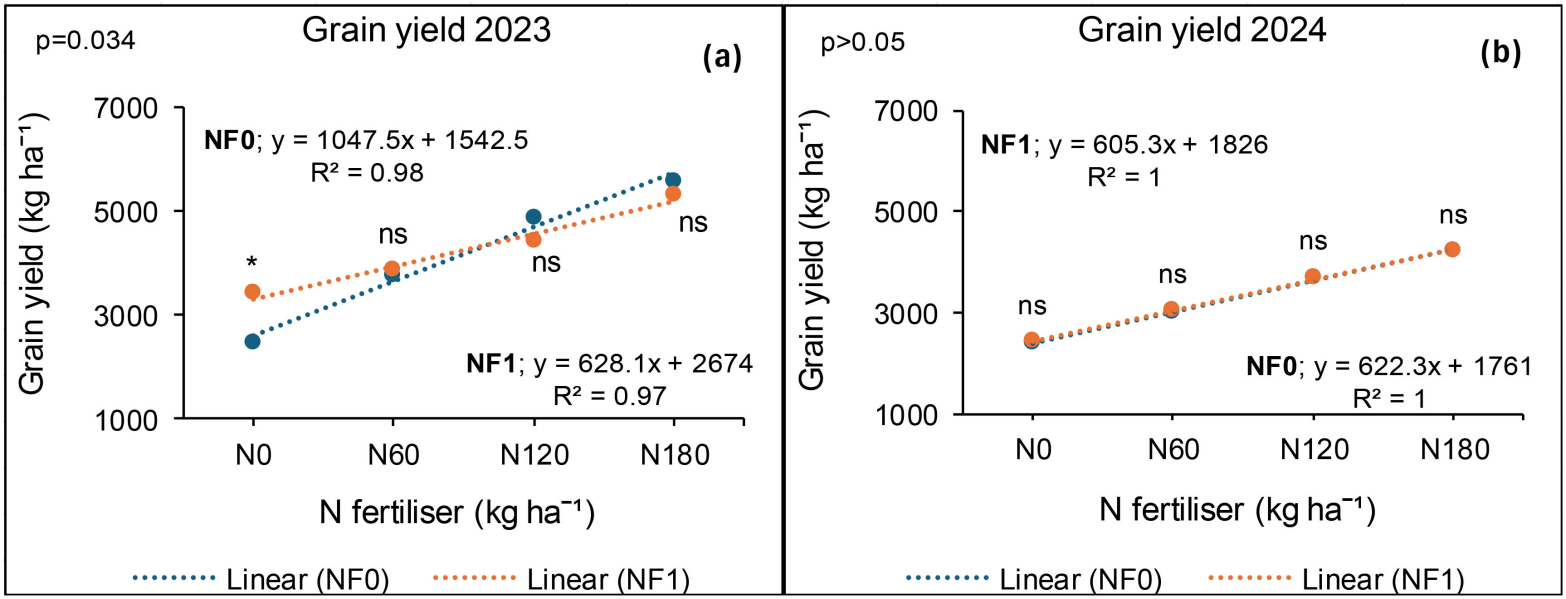
Interactive effects of combined nano Zn and Cu and N fertiliser on grain yield for 2023 **(a)** and 2024 **(b)** at Ofcolaco. NF_0_: no combined nano Zn and Cu; NF_1_: with combined nano Zn and Cu.

During the 2023 season, grain yield was significantly affected by cover crop and N fertiliser application but nano Zn and Cu and all interactions were not significant. A 23% higher grain yield was recorded under cover crop incorporation relative to no application (**Figure 5a**). Nitrogen fertiliser rate of 180 kg ha^−1^ increased grain yield by 44% relative to zero application and 29% compared with the average 60 and 120 kg N ha^−1^, respectively (**Figure 5b**). Moreover, the grain yield obtained under 120 kg N ha^−1^ constituted 85% that of 180 kg N ha^−1^. Furthermore, without the nano Zn and Cu, grain yield was 127% higher under 180 kg N ha^−1^ relative to zero application and 15% higher compared with 120 kg N ha^−1^. Grain yield under 180 kg N ha^−1^ was similar to that of 120 kg N ha^−1^ (**Figure 6a**). However, applying combined nano Zn and Cu increased grain yield under 180 kg N ha^−1^ by 46% compared with the average of 0 and 60 kg N ha^−1^ and 20% relative to 120 kg N ha^−1^ (**Figure 6a**). Nonetheless, grain yield under 120 kg N ha^−1^ was statistically comparable with that of 180 kg N ha^−1^.

In 2024, grain yield was significantly affected by cover crop and N fertiliser, whereas combined nano Zn and Cu had no impact. An interaction effect was only observed between combined nano Zn and Cu and N fertiliser. **Figure 5a** shows that grain yield improved by 43% in plots that had cover crop compared to those without it. Furthermore, N fertiliser applied at 180 kg N ha^−1^, increased grain yield by 76% relative to the unfertilised plots, and by 26% compared with the average of 60 and 120 kg N ha^−1^ (**Figure 5b**). On the other hand, without combined nano Zn and Cu, N fertiliser at 180 kg N ha^−1^ resulted in 78% and 27% higher grain yields compared with 0 kg N ha^−1^ and the average of 60 and 120 kg N ha^−1^ applications, respectively (**Figure 6b**). In addition, nano Zn and Cu application with 180 kg N ha^−1^ resulted in 74% higher grain yield relative to zero application and 25% relative to the average of 60 and 120 kg N ha^−1^ (**Figure 6b**). Overall, the grain yield produced under 120 kg N ha^−1^ was 87% and 88% that of 180 kg N ha^−1^ with and without the application of nano Zn and Cu, respectively.

### Seed crude oil concentration at Syferkuil

**Figure 7:**
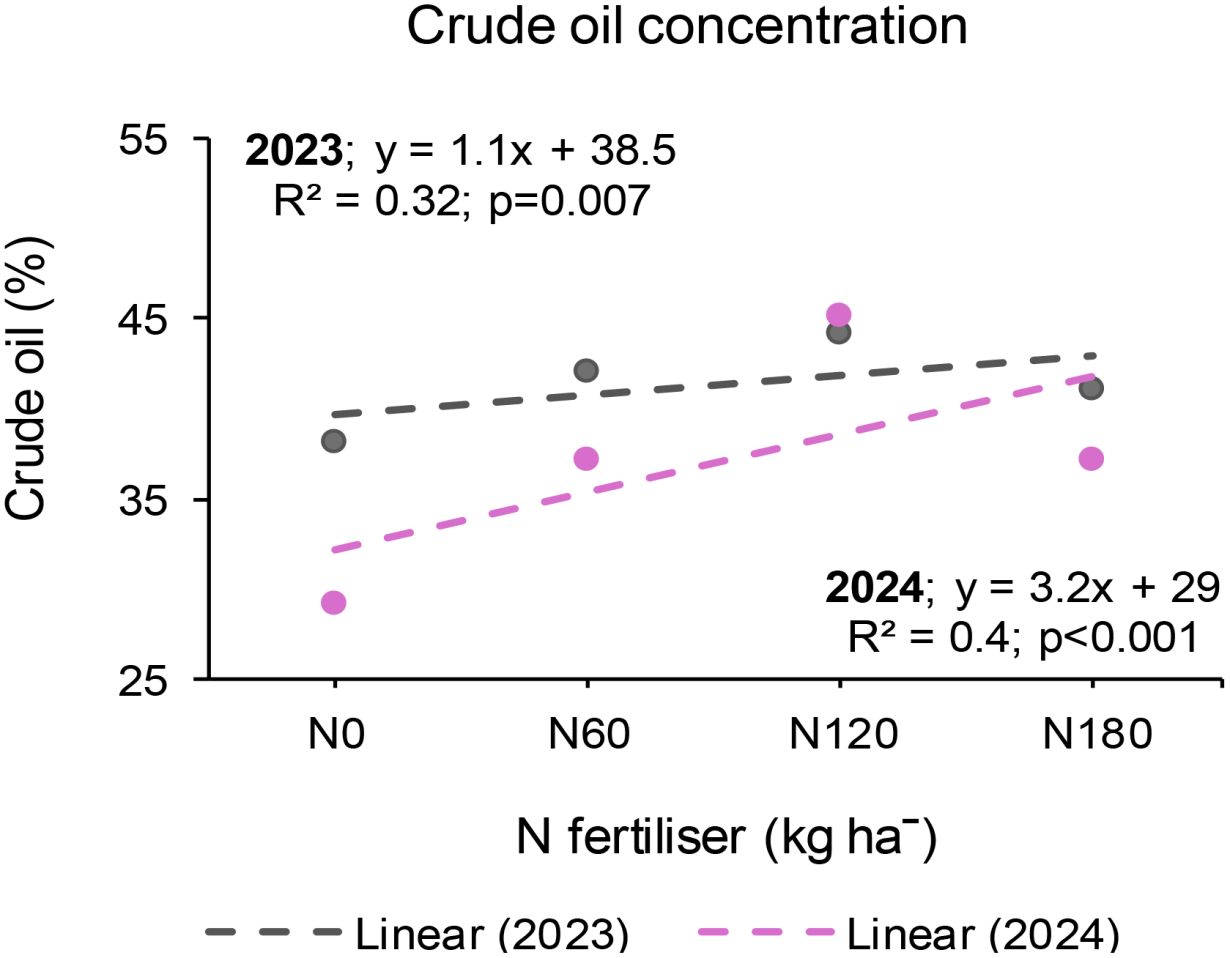
Effect of N fertiliser on seed crude oil concentration at Syferkuil during the 2023 and 2024 seasons.

The seed crude oil concentration at Syferkuil was not influenced by any of the treatments or their interactions in 2023. Irrespective of the treatments, the crude oil ranged from 35% to 46%. In 2024, the seed crude oil concentration was significantly affected by N fertiliser, whereas cover crop, nano Zn and Cu, and the respective interactions had no impact. Nitrogen fertiliser application increased crude oil by 32% under 120 kg N ha^−1^ relative to the average of 0, 60, and 180 kg N ha^−1^ (**Figure 7**). The overall crude oil concentration ranged from 30% to 49% across treatments.

### Seed crude oil concentration at Ofcolaco

**Figure 8:**
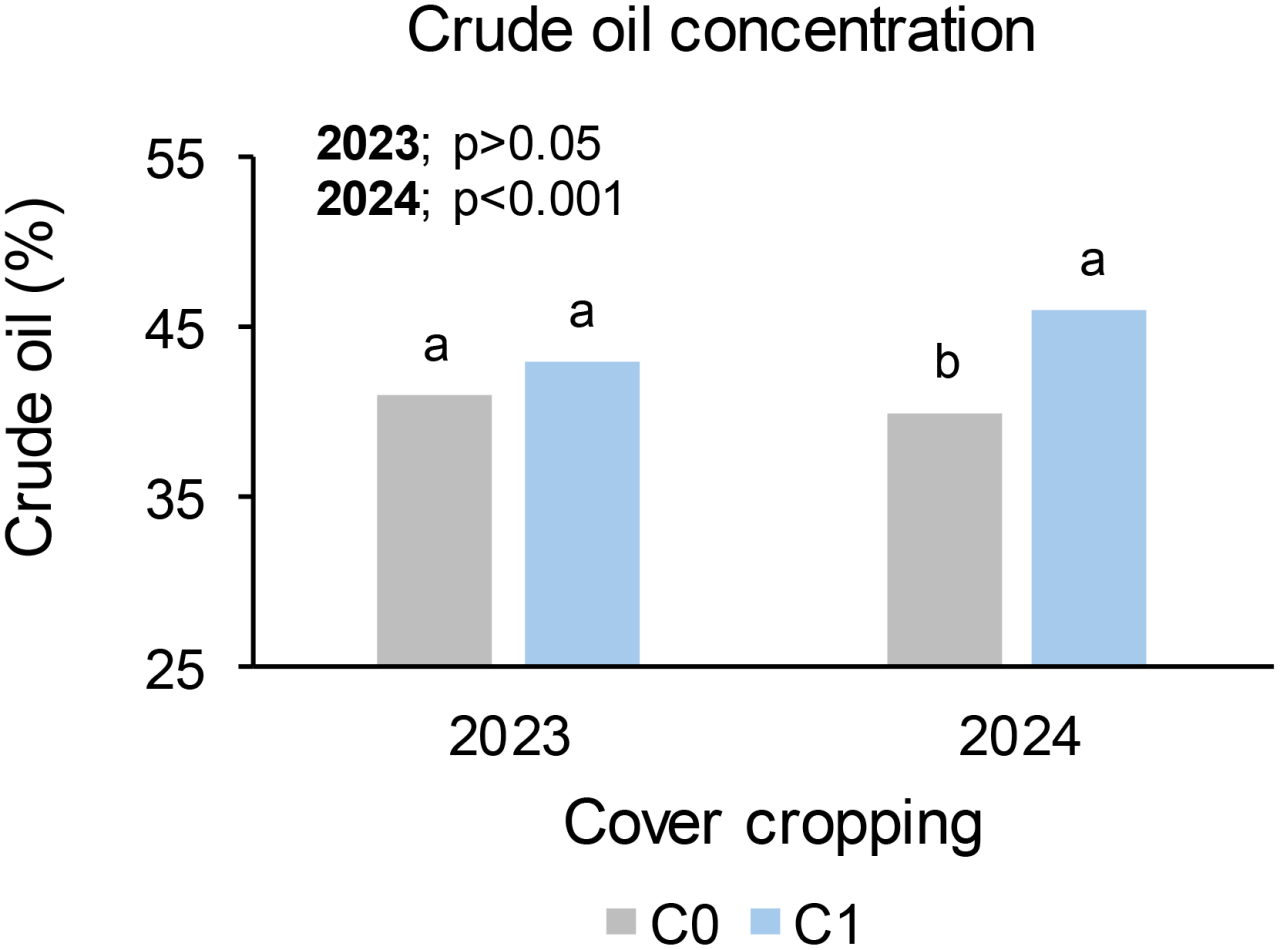
Effect of cover crop on crude oil at Ofcolaco for the 2023 and 2024. C_0_: without cover crop; C_1_: with cover crop.

The seed crude oil concentration at Ofcolaco was not affected by any treatment or their interactions during in 2023. Crude oil concentrations ranged from 37% to 46% across all treatments. In 2024, the seed crude oil was significantly affected by cover crop, whereas combined nano Zn and Cu, N fertiliser, and all interactions were not significant. Incorporating cover crop increased crude oil by 15% compared with treatments without cover crop (**Figure 8**). Furthermore, crude oil ranged from 37% to 49% across all treatments.

### Seed oil yield at Syferkuil

**Figure 9:**
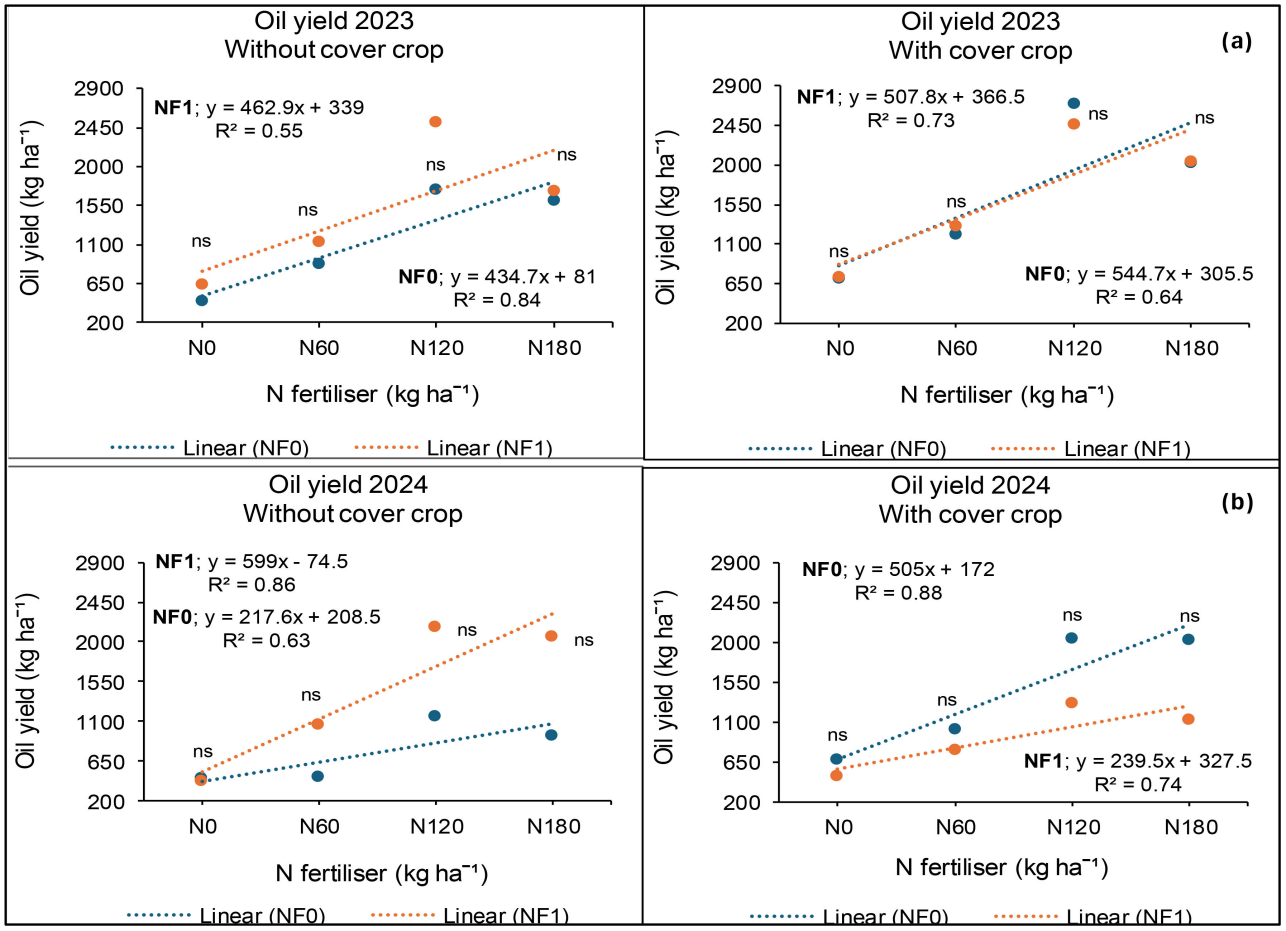
Interaction effects of cover crop, combined nano Zn and Cu and N fertiliser on oil yield at Syferkuil for 2023 **(a)** and 2024 **(b)**. NF_0_: no combined nano Zn and Cu; NF_1_: with combined nano Zn and Cu.

**Figure 10:**
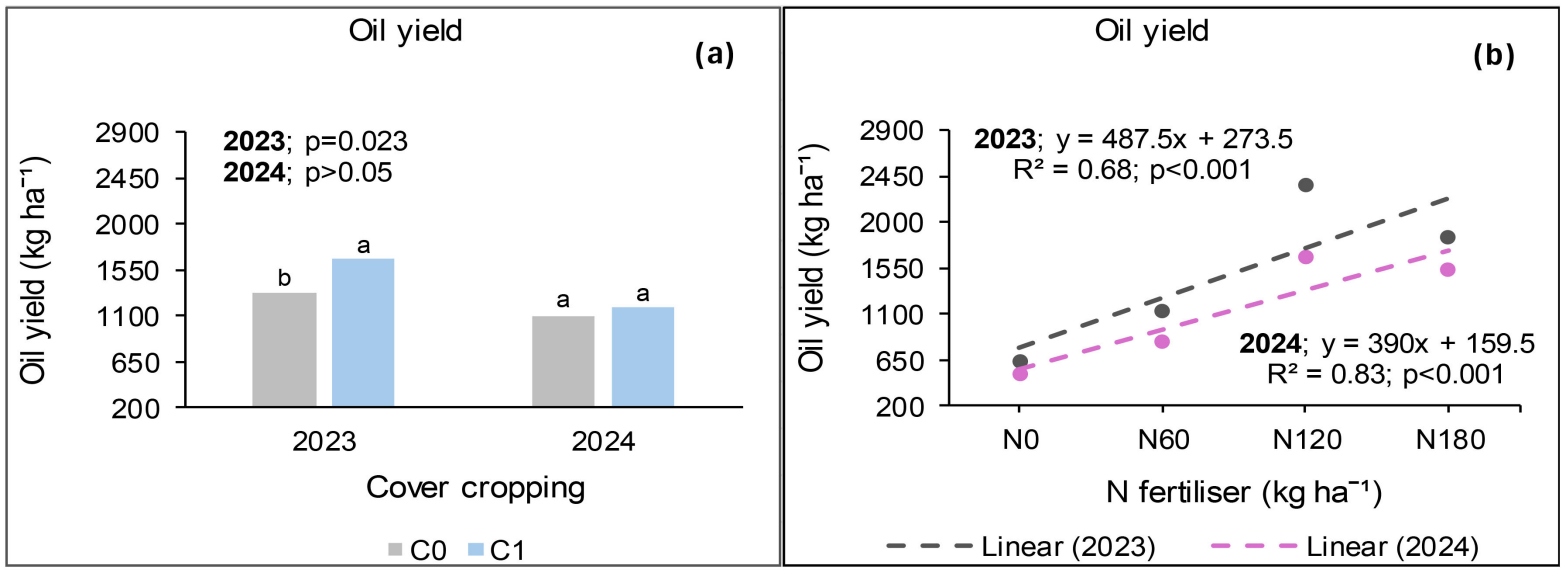
Effects of cover crop and N fertiliser on oil yield at Syferkuil for the 2023 **(a)** and 2024 **(b)**. C_0_: without cover crop; C_1_: with cover crop.

The results from Syferkuil revealed that seed oil yield was significantly affected by cover crop and N fertiliser but not nano Zn and Cu in 2023. The three-factor interactions were not significant (**Figure 9a**). Cover crop incorporation increased oil yield by 24% compared with treatments without cover crop (**Figure 10a**). Nitrogen fertiliser at 120 kg N ha^−1^ also resulted in 27% higher oil yield relative to 180 kg N ha^−1^ and 167% compared with the average of 0 and 60 kg N ha^−1^ (**Figure 10b**).

During 2024, seed oil yield was significantly affected by N fertiliser, whereas cover crop and combined nano Zn and Cu had no effect. Significant interactions were also observed between cover crop and nano Zn and Cu and N fertiliser. Seed oil yield under 120 kg N ha^−1^ was 9% higher, relative to 180 kg N ha^−1^ and 148% greater than the average of 0 and 60 kg N ha^−1^ (**Figure 10b**). Additionally, without cover crop and combined nano Zn and Cu, the application of 120 kg N ha^−1^ resulted in 23% higher oil yield compared with 180 kg N ha^−1^ and 153% relative to the average of 0 and 60 kg N ha^−1^ (**Figure 9b**). Without cover crop only, combined nano Zn and Cu application at 120 kg N ha^−1^ resulted in 192% higher seed oil yield compared with the average of 0 and 60 kg N ha^−1^ and 5% higher than 180 kg N ha^−1^. With cover crop, seed oil yield also increased by 141% under 120 kg N ha^−1^ relative to the average of 0 and 60 kg N ha^−1^ in the absence of nano Zn and Cu. When cover crop and nano Zn and Cu were applied with 120 kg N ha^−1^, seed oil yield increased by 164% compared with 0 kg N ha^−1^ and 17% relative to 180 kg N ha^−1^ (**Figure 9b**). Fertiliser rates of 180 and 120 kg N ha^−1^ were comparable across all interactions.

### Seed oil yield at Ofcolaco

**Figure 11:**
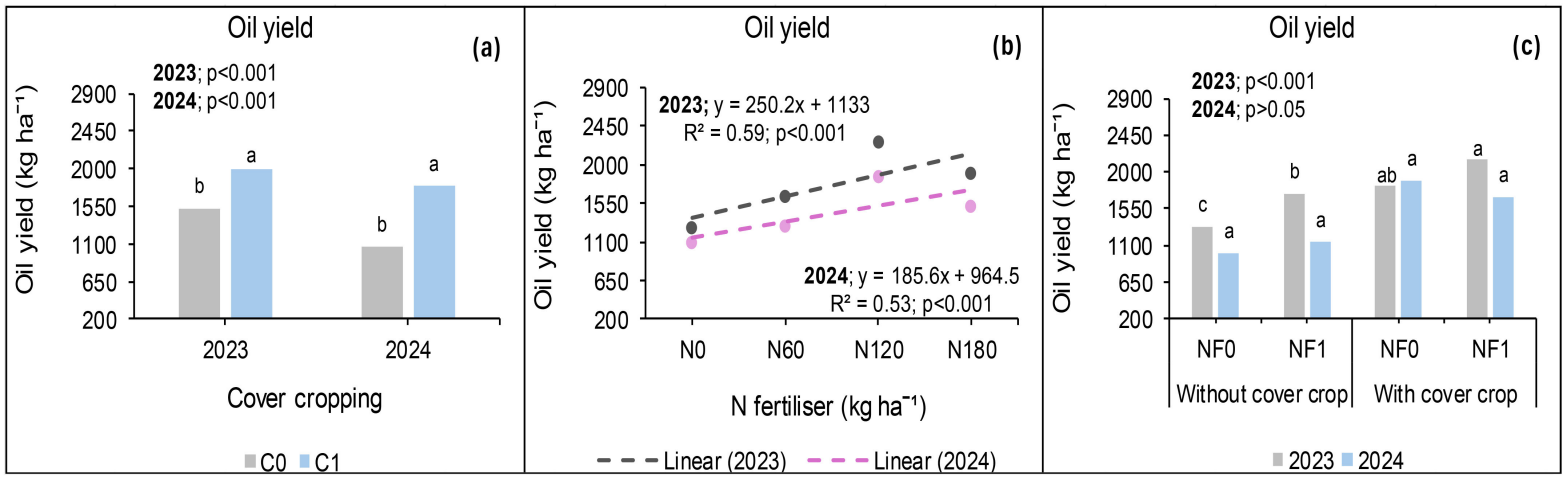
Effects of cover crop **(a)**, N fertiliser **(b)** and the interaction of cover crop and combined nano Zn and Cu **(c)** on oil yield at Ofcolaco for the 2023 and 2024. NF_0_: without combined nano Zn and Cu; Nf_1_: with combined nano Zn and Cu.

The oil yield at Ofcolaco was significantly affected by cover crop and N fertiliser, but not combined nano Zn and Cu during the 2023 growing season. No interaction effects were observed except for cover crop and nano Zn and Cu. Cover crop incorporation increased oil yield by 31% relative to no cover crop (**Figure 11a**). On the other hand, 180 kg N ha^−1^ application resulted in 49% higher oil yield than 0 kg N ha^−1^ but was 16% lower than 120 kg N ha^−1^ (**Figure 11b**). Without cover crop, nano Zn and Cu application increased oil yield by 30% compared with treatments without nanofertiliser (**Figure 11c**). However, there was no difference in oil yield under cover crop incorporation regardless of nanofertiliser application.

The 2024 results indicated significant impacts of cover crop and N fertiliser on oil yield, whereas combined nano Zn and Cu and all interactions exhibited no effects. Oil yield under cover crop incorporation increased by 68% compared with treatments without cover crop (**Figure 11a**). Furthermore, 180 kg N ha^−1^ resulted in 28% higher oil yield compared with the average of 0 and 60 kg N ha^−1^, but 18% lower than 120 kg N ha^−1^ (**Figure 11b**). The application of 180 kg N ha^−1^ was comparable with 60 kg N ha^−1^, whereas 120 kg N ha^−1^ was the highest.

### Seed protein yield at *Syferkuil*

**Figure 12:**
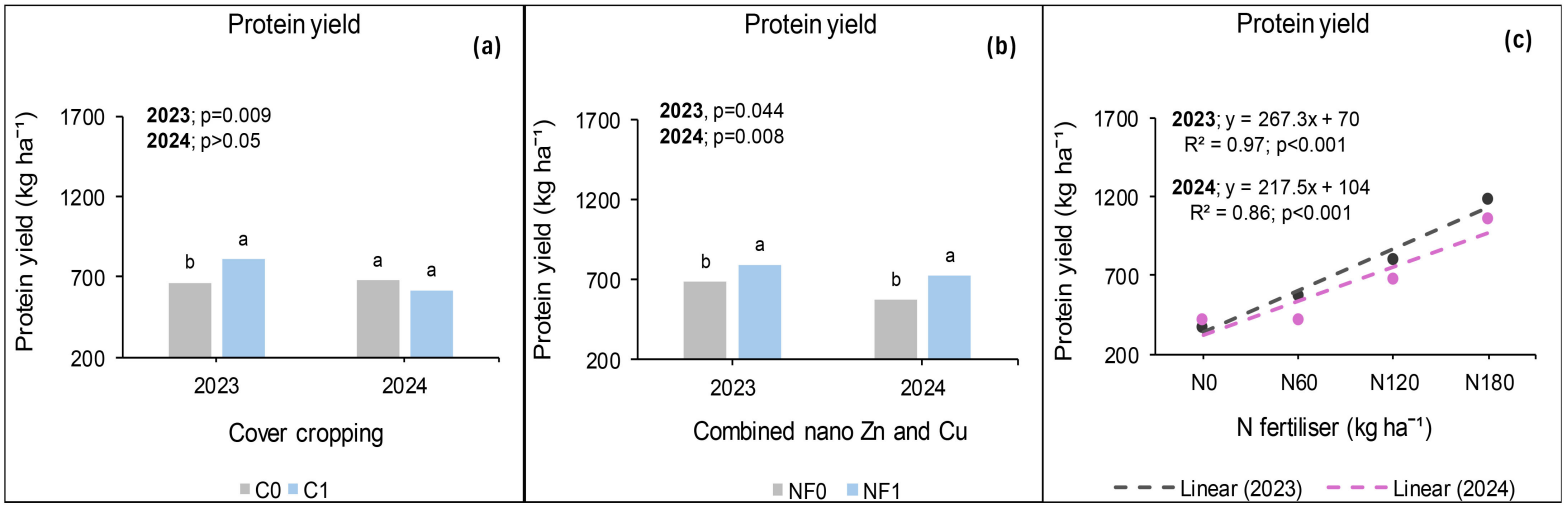
Effects of cover crop **(a)**, combined nano Zn and Cu **(b)** and N fertiliser **(c)** on protein yield at Syferkuil for 2023 and 2024. NF_0_: no combined nano Zn and Cu; NF_1_: with combined nano Zn and Cu.

Canola seed protein yield was calculated from seed protein concentration which ranged from 13% to 31% across treatments, locations, and seasons. In 2023, protein yield was significantly affected by cover crop, combined nano Zn and Cu, and N fertiliser. The interaction of cover crop, combined nano Zn and Cu, and N fertiliser during this season was not significant. Treatments with cover crop produced 21% higher protein yield than those without (**Figure 12a**). Nano Zn and Cu application also increased protein yield by 16% relative to treatments without nanofertiliser (**Figure 12b**). Protein yield also increased by 45% under 180 kg N ha^−1^ of fertiliser application compared with 120 kg N ha^−1^; and 150% relative to the average of 0 and 60 kg N ha^−1^ (**Figure 12c**).

**Figure 13:**
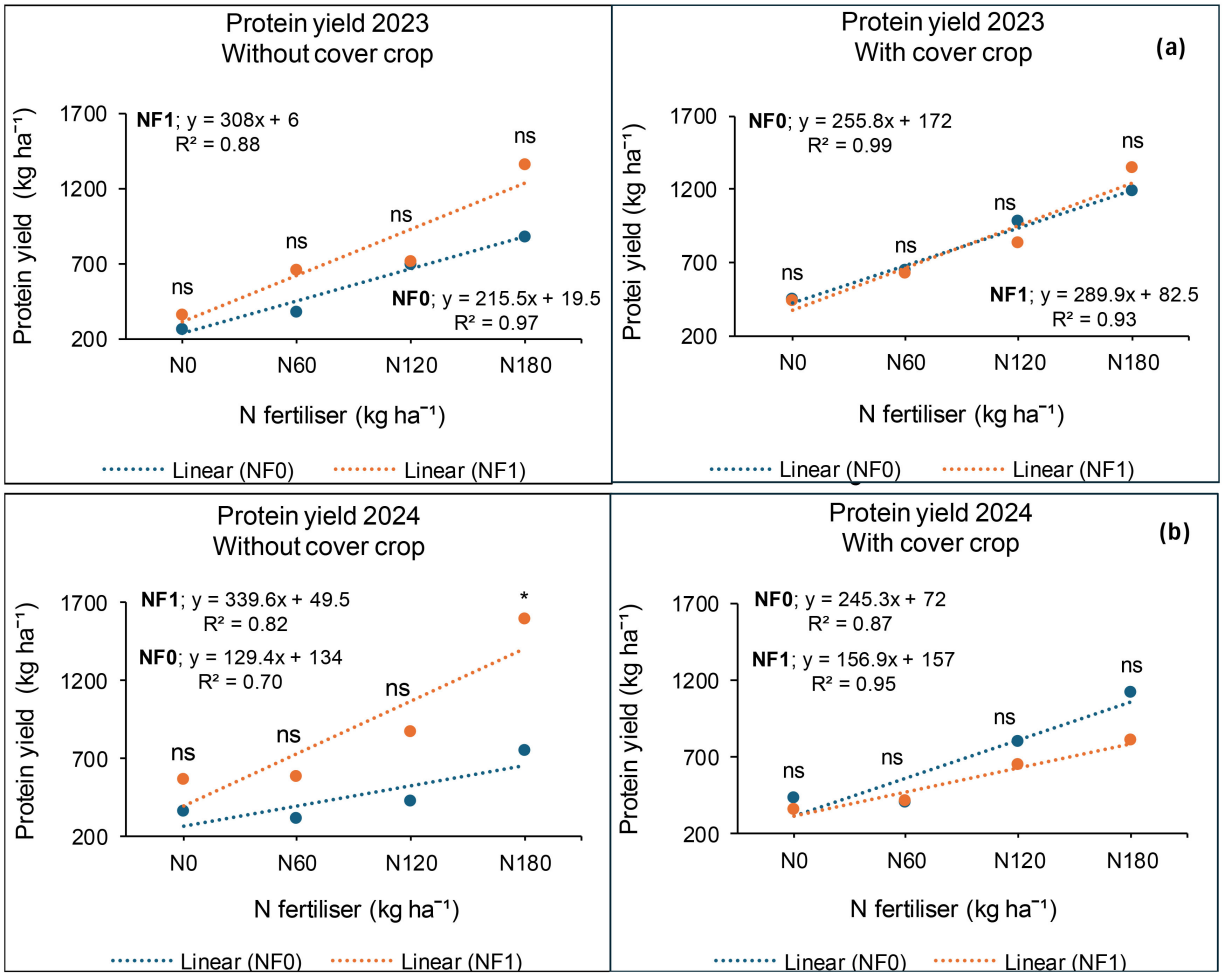
Effects of cover crop, nano Zn and Cu, and N fertiliser on protein yield at Syferkuil for 2023 **(a)** and 2024 **(b)**. NF_0_: no combined nano Zn and Cu; NF_1_: with combined nano Zn and Cu.

During the 2024 season, seed protein yield was significantly influenced by nano Zn and Cu and N fertiliser. Significant interactive effects of cover crop, combined nano Zn and Cu and N fertiliser were also observed on the parameter. A 27% higher protein yield was recorded under nano Zn and Cu compared with no application (**Figure 12b**). Nitrogen fertiliser application at 180 kg N ha^−1^ resulted in 57% and 151% increases in protein yield compared with 120 and 0 kg N ha^−1^, respectively (**Figure 12c**). The three-factor interaction showed no variation in protein yield in the absence of cover crop and combined nano Zn and Cu (**Figure 13b**). However, with nano Zn and Cu an increase of 85% and 138% was found at 180 kg N ha^−1^ compared with 120 and 0 kg N ha^−1^, respectively, without cover crop. With cover crop, protein yield was recorded at 40% and 159% under 180 kg N ha^−1^, respectively, compared with 120 and 0 kg N ha^−1^ without the application of nano Zn and Cu. In contrast, the interaction with cover crop, combined nano Zn and Cu application, had no variation regardless of N fertiliser. Protein concentrations for 2024 ranged from 13% to 30% across all treatments (**Figure 13b**).

### Seed protein yield at Ofcolaco

**Figure 14:**
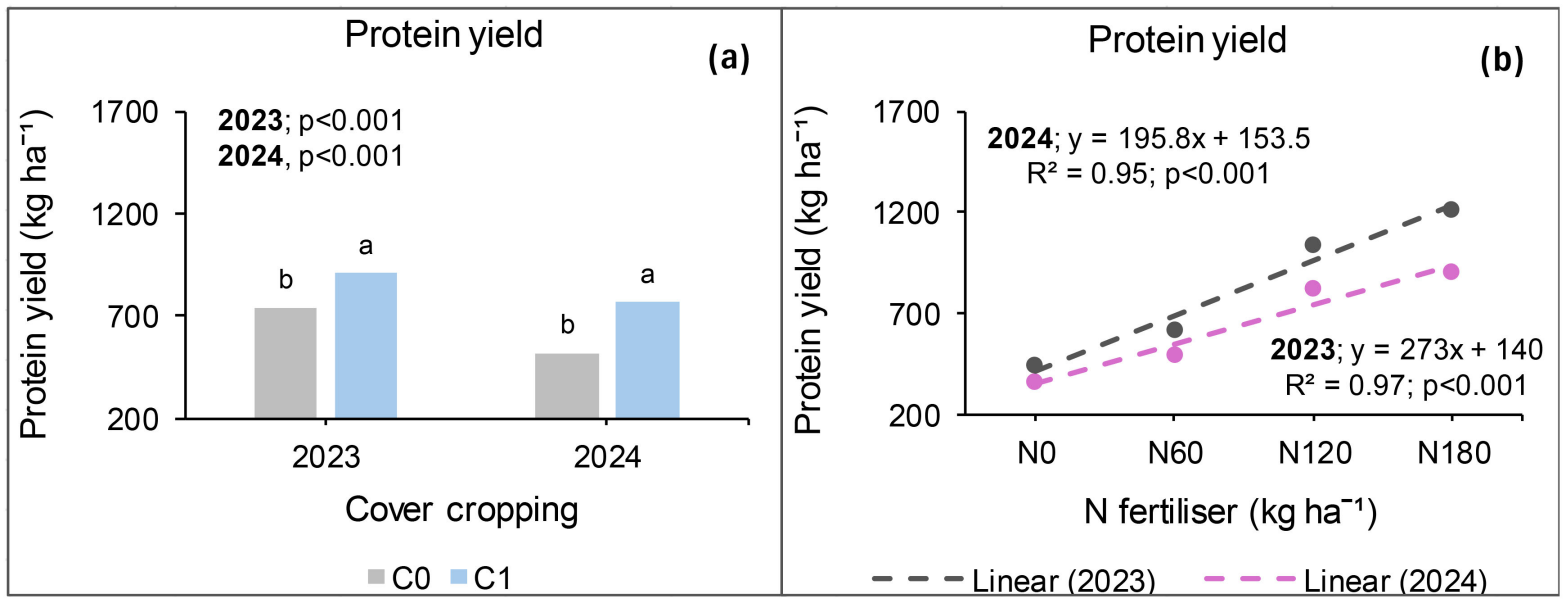
Effects of cover crop **(a)** and N fertiliser **(b)** on protein yield at Ofcolaco for the 2023 and 2024. C_0_: without cover crop; C_1_: with cover crop.

Significant effects of cover crop and N fertiliser were observed on protein yield in 2023, whereas combined nano Zn and Cu had no impact. Interactive effects were only significant for cover crop and combined nano Zn and Cu. A 23% increase in protein yield was recorded under cover crop incorporation compared with treatments without it (**Figure 14a**). Additionally, 16% and 176% increases in protein yield occurred under 180 kg N ha^−1^ relative to 120 and 0 kg N ha^−1^, respectively (**Figure 14b**). In 2024, the results revealed significant effects of cover crop and N fertiliser on protein yield whereas combined nano Zn and Cu and all interactions had no impact. During this season, a 47% protein accumulation was observed under cover crop incorporation relative to treatments with it (**Figure 14a**). Also, protein yield under 180 kg N ha^−1^ was 11% and 152% higher compared with the application of 120 and 0 kg N ha^−1^.

### Yield components and relationships at Syferkuil

#### Number of pods per plant

The number of pods per plant during the 2023 and 2024 seasons was significantly affected by N fertiliser but not cover crop and combined nano Zn and Cu. Interactive effects were not significant in both seasons, with the exception of cover crop and combined nano Zn and Cu in 2024. Therefore, applying 180 kg N ha^−1^ resulted in 47% and 98% increase in the number of pods per plant compared with 120 and 0 kg N ha^−1^, respectively, during 2023 (**Table 2**). Meanwhile in 2024, the number of pods per plant at 180 kg N ha^−1^ was 29% and 84% higher relative to 120 and 0 kg N ha^−1^ (**Table 2**). Furthermore, the application of nano Zn and Cu without cover crop did not affect the number of pods per plant. However, combining cover crop with nano Zn and Cu application reduced the number of pods per plant by 8% compared with no nanofertiliser application (**Table 2**).

**Table 2:** Treatment and interaction effects on yield components in canola production at Syferkuil.

Treatments	2023			2024		
	Number of pods per plant	TSW (g)	Number of seeds per pod	Number of pods per plant	TSW (g)	Number of seeds per pod
C_0_	256	4.48	20.9	224	2.74	23
C_1_	255	4.46	20.8	236	2.75	23
*p <0.05*	ns	ns	ns	ns	ns	ns
NF_0_	250	4.75^a^	20.6	225	2.66	22
NF_1_	262	4.20^b^	21.2	235	2.83	24
*p <0.05*	ns	*	ns	ns	ns	ns
N_0_	167^d^	3.37^c^	18.4^c^	169^c^	2.00^b^	20^c^
N_60_	231^c^	4.06^bc^	19.8^c^	199^b^	2.38^b^	22^b^
N_120_	295^b^	4.55^b^	21.5^b^	241^a^	3.22^a^	25^a^
N_180_	330^a^	5.90^a^	23.9^a^	311^a^	3.39^a^	27^a^
*p <0.05*	***	***	***	***	***	***
C_0_NF_0_	242	4.58	21.2	203^b^	2.17	21^b^
C_0_NF_1_	270	4.38	20.8	244^ab^	3.32	26^a^
C_1_NF_0_	257	4.92	20.1	246^a^	3.16	25^a^
C_1_NF_1_	253	4.01	21.6	226^b^	2.34	21^b^
*p <0.05*	ns	ns	ns	***	ns	***
C_0_N_0_	163	3.61	18.7	164	1.87	20
C_0_N_60_	238	4.22	19.7	186	2.4	22
C_0_N_120_	293	4.67	21.7	237	3.18	25
C_0_N_180_	331	5.41	23.8	309	3.53	27
C_1_N_0_	172	3.14	18.2	174	2.12	19
C_1_N_60_	224	3.91	19.8	213	2.37	22
C_1_N_120_	296	4.42	21.3	246	3.26	26
C_1_N_180_	329	6.39	24.0	313	3.25	27
*p <0.05*	ns	ns	ns	ns	ns	ns
NF_0_N_0_	164	3.98	18.7	161	2.06	19
NF_0_N_60_	225	4.02	19.0	197	2.34	22
NF_0_N_120_	286	4.85	21.7	236	3.05	24
NF_0_N_180_	324	6.14	22.8	305	3.21	27
NF_1_N_0_	171	2.77	18.2	177	1.95	20
NF_1_N_60_	237	4.11	20.2	202	2.42	22
NF_1_N_120_	303	4.24	21.3	247	3.39	27
NF_1_N_180_	336	5.66	25.0	316	3.57	27
*p <0.05*	ns	ns	ns	ns	ns	ns
C_0_NF_0_N_0_	153	4.42	19.3	146	1.61^d^	17
C_0_NF_0_N_60_	231	3.60	19.3	171	2.11^d^	20
C_0_NF_0_N_120_	282	4.92	22.3	214	2.32^d^	21
C_0_NF_0_N_180_	303	5.37	23.7	282	2.63^cd^	25
C_0_NF_1_N_0_	174	2.80	18.0	181	2.13^d^	23
C_0_NF_1_N_60_	246	4.83	19.3	200	2.68^bcd^	24
C_0_NF_1_N_120_	303	4.42	21.0	260	4.04^a^	29
C_0_NF_1_N_180_	357	5.46	22.0	335	4.43^a^	28
C_1_NF_0_N_0_	174	3.54	18.3	175	2.50^d^	21
C_1_NF_0_N_60_	219	4.43	18.3	222	2.57^d^	23
C_1_NF_0_N_120_	288	4.78	20.3	258	3.78^abc^	27
C_1_NF_0_N_180_	345	6.92	21.0	328	3.79^ab^	28
C_1_NF_1_N_0_	168	2.73	18.3	172	1.76^d^	17
C_1_NF_1_N_60_	228	3.38	20.3	203	2.16^d^	20
C_1_NF_1_N_120_	303	4.07	21.7	233	2.73^bcd^	24
C_1_NF_1_N_180_	312	5.87	26.0	297	2.70^bcd^	25
*p <0.05*	ns	ns	ns	ns	**	ns

Significance levels: *p <0.05; **p <0.01; ***p <0.001; ns means not significant: >0.05. Means separated by different letters in each column are significantly different. ¥ *C0, without cover crop; C1, with cover crop;*¥ *NF0, without combined nano Zn and Cu; NF1, with combined nano Zn and Cu.*

#### Thousand seed weight

The findings at Syferkuil revealed significant effects of combined nano Zn and Cu and N fertiliser on thousand seed weight (TSW) in 2023, whereas cover crop and all interactions had no effect. As such combined nano Zn and Cu reduced the TSW by 12% compared with no application (**Table 2**). Meanwhile, N fertiliser resulted in 30% and 75% increase in TSW under 180 kg N ha^−1^ compared with 120 and 0 kg N ha^−1^, respectively (**Table 2**).

In 2024, TSW was significantly affected by N fertiliser, but cover crop and combined nano Zn and Cu were not. Interactive effects were also observed for cover crop, nano Zn and Cu, and N fertiliser only. The application of 180 kg N ha^−1^ resulted in a 55% increase in TSW compared with the average of 0 and 60 kg N ha^−1^ applications (**Table 2**). Furthermore, without the concurrent application of cover crop and nano Zn and Cu, the nitrogen fertiliser effect on TSW was not significant (**Table 2**). However, with sole application of nano Zn and Cu, TSW was increased by 108% at 180 kg N ha^−1^ relative to 0 kg N ha^−1^ in the absence of a cover crop. With cover crop incorporation, TSW was similar between 180 and 120 kg N ha^−1^ applications, but there was a 52% increase relative to 0 kg N ha^−1^ (**Table 2**). Conversely, when cover crop was combined with nano Zn and Cu, TSW was irresponsive, irrespective of N fertiliser application.

#### Number of seeds per pod

The number of seeds per pod in 2023 and 2024 was significantly affected by N fertiliser, whereas cover crop, combined nano Zn and Cu and all interactions had no impact. An increase of 11% and 30% in the number of seeds per pod occurred at 180 kg N ha^−1^ relative to 120 and 0 kg N ha^−1^, respectively (**Table 2**). In 2024, an increase of 8% and 35% in seed count per pod was recorded under 180 kg N ha^−1^ relative to 120 and 0 kg N ha^−1^, respectively (**Table 2**). Additionally, the number of seeds per pod at 180 kg N ha^−1^ was similar to that at 120 kg N ha^−1^. Without cover crop, the application of combined nano Zn and Cu resulted in a 24% increase in the number of seeds per pod relative to treatments lacking nanofertiliser. When cover crop was incorporated, there was a 16% reduction in the number of seeds per pod under the application of combined nano Zn and Cu compared with plots without it (**Table 2**).

### Relationship between grain yield and yield components at Syferkuil

**Figure 15:**
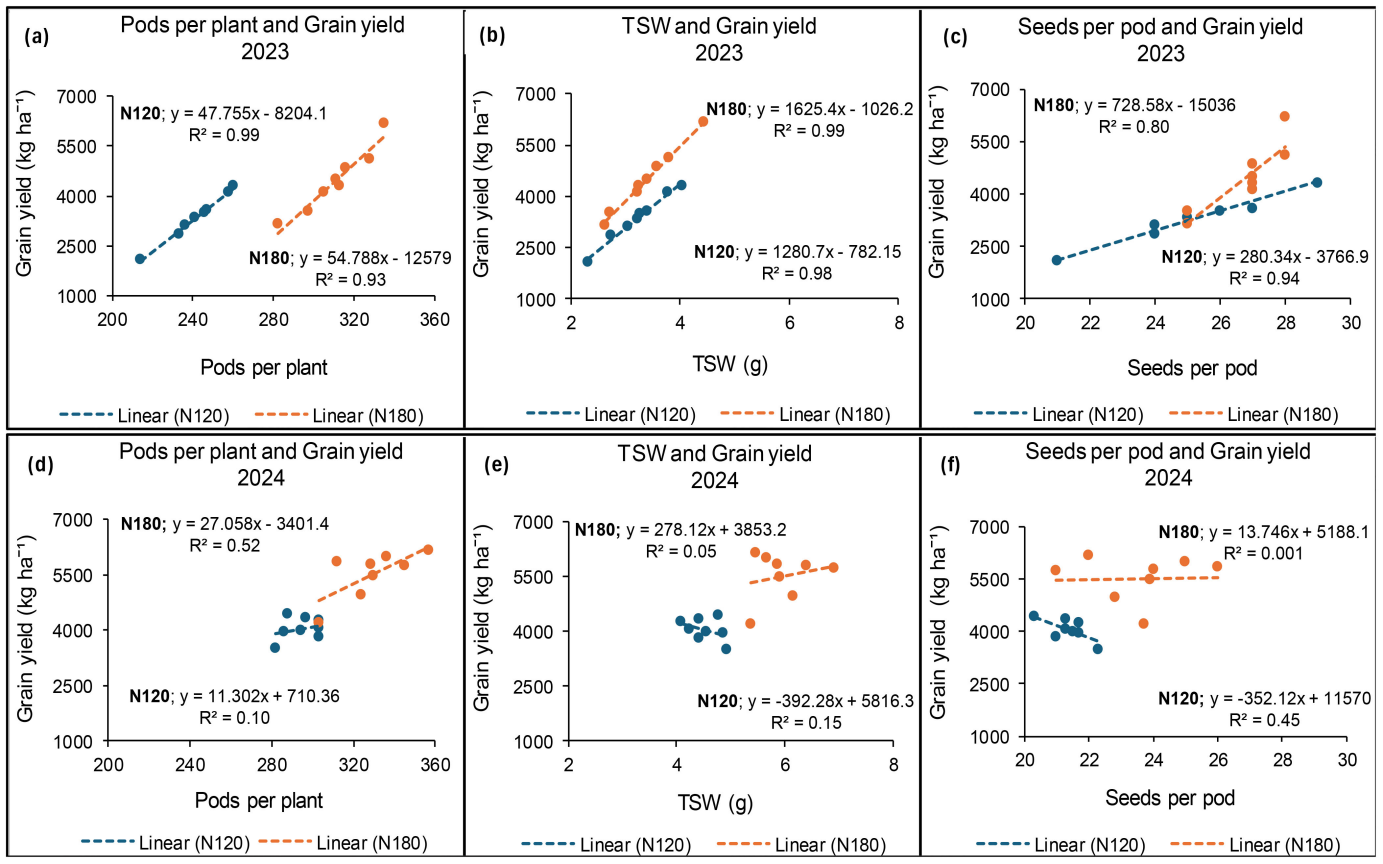
Relationship between grain yield and pods per plant **(a, d)**, TSW **(b, e)** and seeds per pod **(c, f)** under N fertiliser application at Syferkuil in 2023 and 2024.

In 2023, N fertiliser rates of 120 and 180 kg N ha^−1^ showed strong positive correlations between grain yield and the number of pods per plant, TSW, and the number of seeds per pod. The linear regression strength for grain yield in relation to pods per plant was found to be 99% and 93% under 120 and 180 kg N ha^−1^, respectively (**Figure 15a**). Grain yield and TSW showed a strength of 98% and 99%, whereas those of seeds per pod were 94% and 80% at 120 and 180 kg N ha^−1^, respectively (**Figures 15b, c**). In contrast, 2024 demonstrated weakened relationships between grain yield and the selected yield components with respect to N fertiliser application. A 52% linear relationship was found between grain yield and pods per plant under 180 kg N ha^−1^, and 45% for grain yield in relation to seeds per pod under 120 kg N ha^−1^ (**Figures 15d, f**). Moreover, a weak relationship was observed between grain yield and TSW as shown by R^2^ = 15% and R^2^ = 5% at 120 and 180 kg N ha^−1^, respectively (**Figure 15e**).

### Yield components and relationships at Ofcolaco

#### Number of pods per plant

During 2023, the number of pods per plant was significantly affected by combined nano Zn and Cu and N fertiliser but not cover crop (**Table 3**). Significant interactions were also observed on the number of pods per plant. As such, a 11% increase in the parameter was recorded under combined nano Zn and Cu relative to no application. On the other hand, N fertiliser application of 180 kg N ha^−1^ resulted in 35% and 81% higher number of pods per plant compared with 120 and 0 kg N ha^−1^, respectively (**Table 3**). Furthermore, without cover crop and combined nano Zn and Cu application, the number of pods per plant under 180 kg N ha^−1^ increased by 34% relative to 120 kg N ha^−1^ and 113% compared with 0 kg N ha^−1^, respectively. Meanwhile, the application of nano Zn and Cu under no cover crop increased the number of pods per plant by 56% and 114% at 180 kg N ha^−1^ compared with 120 and 0 kg N ha^−1^, respectively (**Table 3**). With cover crop incorporation, the number of pods per plant was comparable for all application rates without nano Zn and Cu. However, when cover crop and combined nano Zn and Cu were incorporated, the number of pods per plant was 31% and 95% higher under 180 kg N ha^-1^ compared with 120 and 0 kg N ha^−1^, respectively (**Table 3**).

**Table 3:** Treatment and interaction effects on yield components after canola production at Ofcolaco.

Treatments	2023			2024		
	Number of pods per plant	TSW (g)	Number of seeds per pod	Number of pods per plant	TSW (g)	Number of seeds per pod
C_0_	128	4.53	23^b^	108^b^	2.38	22
C_1_	121	4.39	25^a^	122^a^	2.33	22
*p <0.05*	ns	ns	*	*	ns	ns
NF_0_	118^b^	4.48	24	114	2.36	22
NF_1_	131^a^	4.43	24	115	2.35	22
*p <0.05*	*	ns	ns	ns	ns	ns
N_0_	93^c^	4.36	24	78^d^	2.26	22
N_60_	112^b^	4.64	24	100^c^	2.39	22
N_120_	124^b^	4.39	24	129^b^	2.40	22
N_180_	168^a^	4.44	24	152^a^	2.37	22
*p <0.05*	**	ns	ns	**	ns	ns
C_0_NF_0_	132^ab^	4.56	23	101^b^	2.35	22
C_0_NF_1_	122^c^	4.23	24	115^ab^	2.41	23
C_1_NF_0_	103^c^	4.41	24	128^a^	2.37	22
C_1_NF_1_	140^a^	4.64	25	116^ab^	2.30	22
*p <0.05*	*	ns	ns	*	ns	ns
C_0_N_0_	86^e^	4.00	24	73	2.23	22
C_0_N_60_	112^cde^	4.30	24	94	2.32	21
C_0_N_120_	128^bc^	4.52	23	121	2.44	23
C_0_N_180_	184^a^	4.75	23	144	2.52	22
C_1_N_0_	101^de^	4.72	25	84	2.29	22
C_1_N_60_	113^cde^	4.98	25	106	2.47	22
C_1_N_120_	120^cd^	4.27	24	138	2.36	21
C_1_N_180_	153^b^	4.13	25	159	2.22	23
*p <0.05*	*	ns	ns	ns	ns	ns
NF_0_N_0_	95^d^	4.31	25	81	2.34	22
NF_0_N_60_	112^de^	4.48	23	98	2.41	22
NF_0_N_120_	116^bc^	4.55	23	129	2.32	22
NF_0_N_180_	149^a^	4.58	23	149	2.37	23
NF_1_N_0_	92^d^	4.40	24	76	2.18	22
NF_1_N_60_	113^cd^	4.80	25	102	2.38	21
NF_1_N_120_	132^cd^	4.23	24	129	2.48	22
NF_1_N_180_	188^b^	4.30	25	154	2.37	22
*p <0.05*	*	ns	ns	ns	ns	ns
C_0_NF_0_N_0_	87^f^	3.93	25	72	2.29	22
C_0_NF_0_N_60_	118^def^	4.50	22	86	2.29	22
C_0_NF_0_N_120_	139^cde^	4.43	23	114	2.24	23
C_0_NF_0_N_180_	186^ab^	5.37	22	131	2.57	22
C_0_NF_1_N_0_	85^f^	4.07	23	74	2.17	23
C_0_NF_1_N_60_	106^def^	4.10	24	103	2.36	20
C_0_NF_1_N_120_	117^def^	4.60	24	127	2.64	23
C_0_NF_1_N_180_	182^abc^	4.13	24	156	2.48	21
C_1_NF_0_N_0_	102^def^	4.70	25	90	2.39	23
C_1_NF_0_N_60_	106^def^	4.47	25	110	2.52	22
C_1_NF_0_N_120_	94^f^	4.67	24	143	2.40	22
C_1_NF_0_N_180_	112^def^	3.80	24	167	2.17	23
C_1_NF_1_N_0_	99^ef^	4.73	25	78	2.20	21
C_1_NF_1_N_60_	119^def^	5.50	24	102	2.41	22
C_1_NF_1_N_120_	147^bcd^	3.87	25	132	2.31	20
C_1_NF_1_N_180_	193^a^	4.47	25	151	2.27	23
*p <0.05*	*	ns	ns	ns	ns	ns

Significance levels: *p <0.05; **p <0.01; ***p <0.001; ns means not significant: >0.05; Means separated by different letters in each column are significantly different. ¥ *C0: without cover crop; C1: with cover crop;*¥ NF*0: without combined nano Zn and Cu; NF1: with combined nano Zn and Cu.*

During the 2024 growing season, cover crop and N fertiliser had a significant effect on the number of pods per plant, whereas combined nano Zn and Cu and all interactions had no impact (**Table 3**). A 13% increase in the number of pods per plant was recorded under cover crop incorporation relative to treatments without it (**Table 3**). Moreover, the number of pods per plant increased by 18% and 95% under 180 kg N ha^−1^ application compared with 120 and 0 kg N ha^−1^, respectively.

#### Thousand seed weight

The findings at Ofcolaco indicated that TSW was unaffected by any of the treatment factors or their interactions during the 2023 and 2024 seasons (**Table 3**). However, TSW ranged from 3.80 to 5.37 g in 2023 and 2.17 to 2.58 g in 2024 across all treatments.

#### Number of seeds per pod

The 2023 season revealed significant effects of cover crop on the number of seeds per pod. However, combined nano Zn and Cu, N fertiliser, and all interactions had no impact. The number of seeds per pod under cover crop incorporation was 9% higher compared with no cover crop (**Table 3**). In 2024, the number of seeds per pod at Ofcolaco ranged from 20 to 23, with no response across all treatments.

### Relationship between grain yield and yield components at Ofcolaco

**Figure 16:**
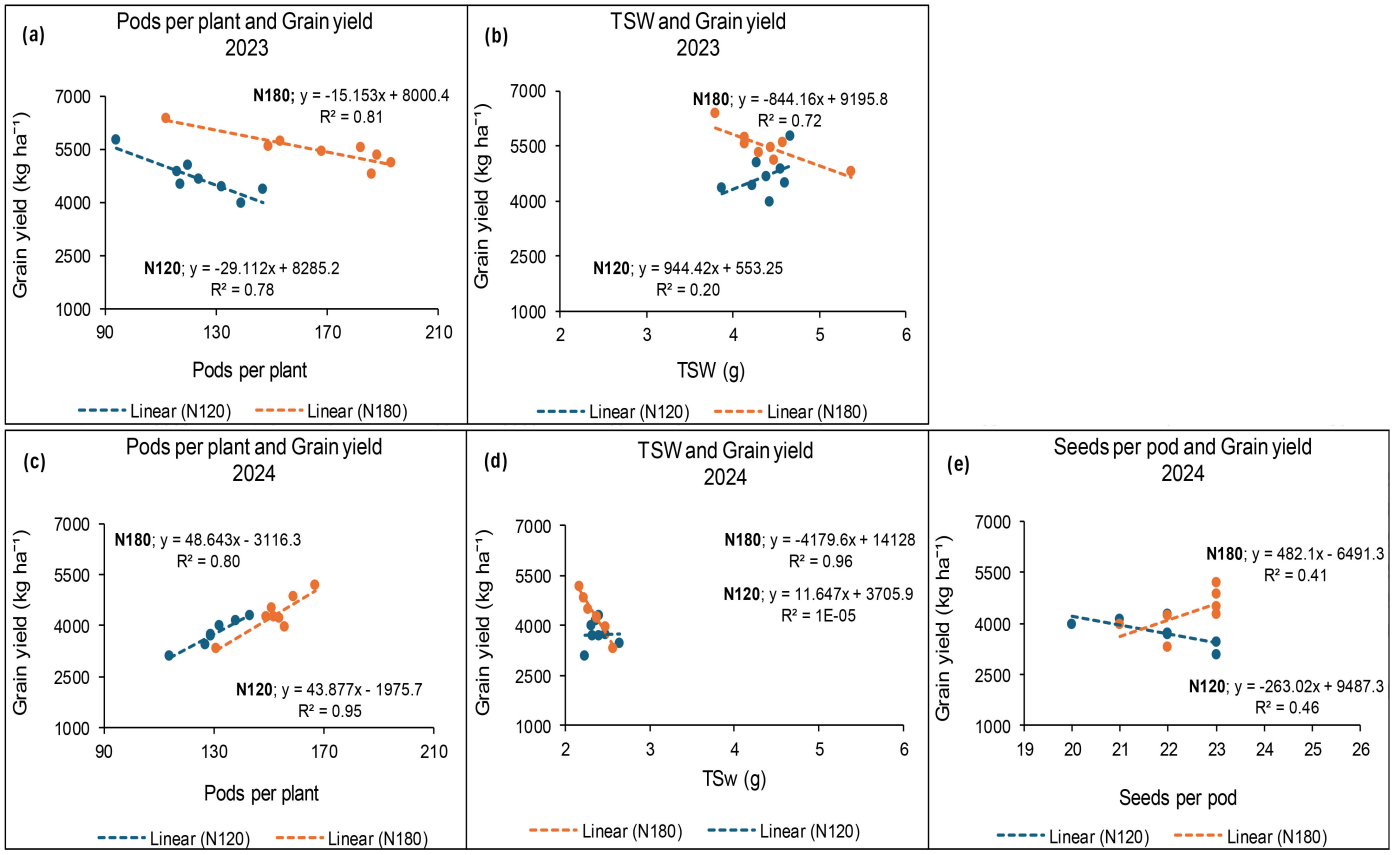
Relationship between grain yield and pods per plant **(a, c)**, TSW **(b, d)** and seeds per pod **(e)** under N fertiliser application at Ofcolaco in 2023 and 2024.

The results from Ofcolaco in 2023 demonstrated a decreasing relation of 78% and 81% between grain yield and number of pods per plant under 120 and 180 kg N ha^−1^, respectively (**Figure 16a**). Conversely, TSW and grain yield exhibited a 72% linear relationship at 180 kg N ha^−1^, whereas a weak positive relation was observed at 120 kg N ha^−1^ (**Figure 16b**). A weak non-linear relationship between grain yield and seeds per pod was observed for 120 (R^2^ = 0.007) and 180 kg N ha^−1^ (R^2^ = 0.08). In 2024, a strong positive relationship was observed between grain yield and pods per plant with values of 95% and 80% at 120 and 180 kg N ha^−1^, respectively (**Figure 16c**). **Figure 16d** illustrates a strong positive relationship (96%) between TSW and grain yield at 180 kg N ha^−1^, whereas a weak relationship was observed at 120 kg N ha^−1^. Additionally, a moderately positive relationship for grain yield and seeds per pod, represented by 46% and 41% at 120 and 180 kg N ha^−1^, respectively (**Figure 16e**).

### Harvest index at *Syferkuil*

**Figure 17:**
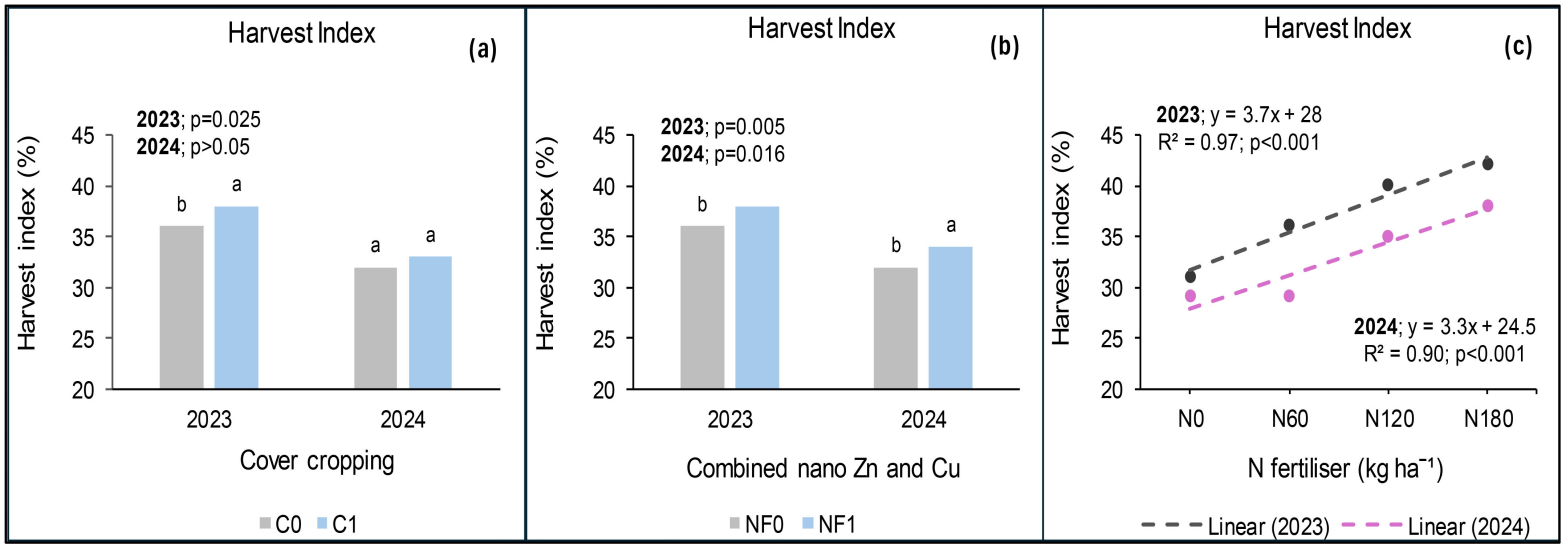
Effects of cover crop **(a)**, combined nano Zn and Cu **(b)**, and N fertiliser **(c)** on HI at Syferkuil for 2023 and 2024. C_0_: without cover crop; C_1_: with cover crop; NF_0_: without combined nano Zn and Cu; NF_1_: with combined nano Zn and Cu.

**Figure 18:**
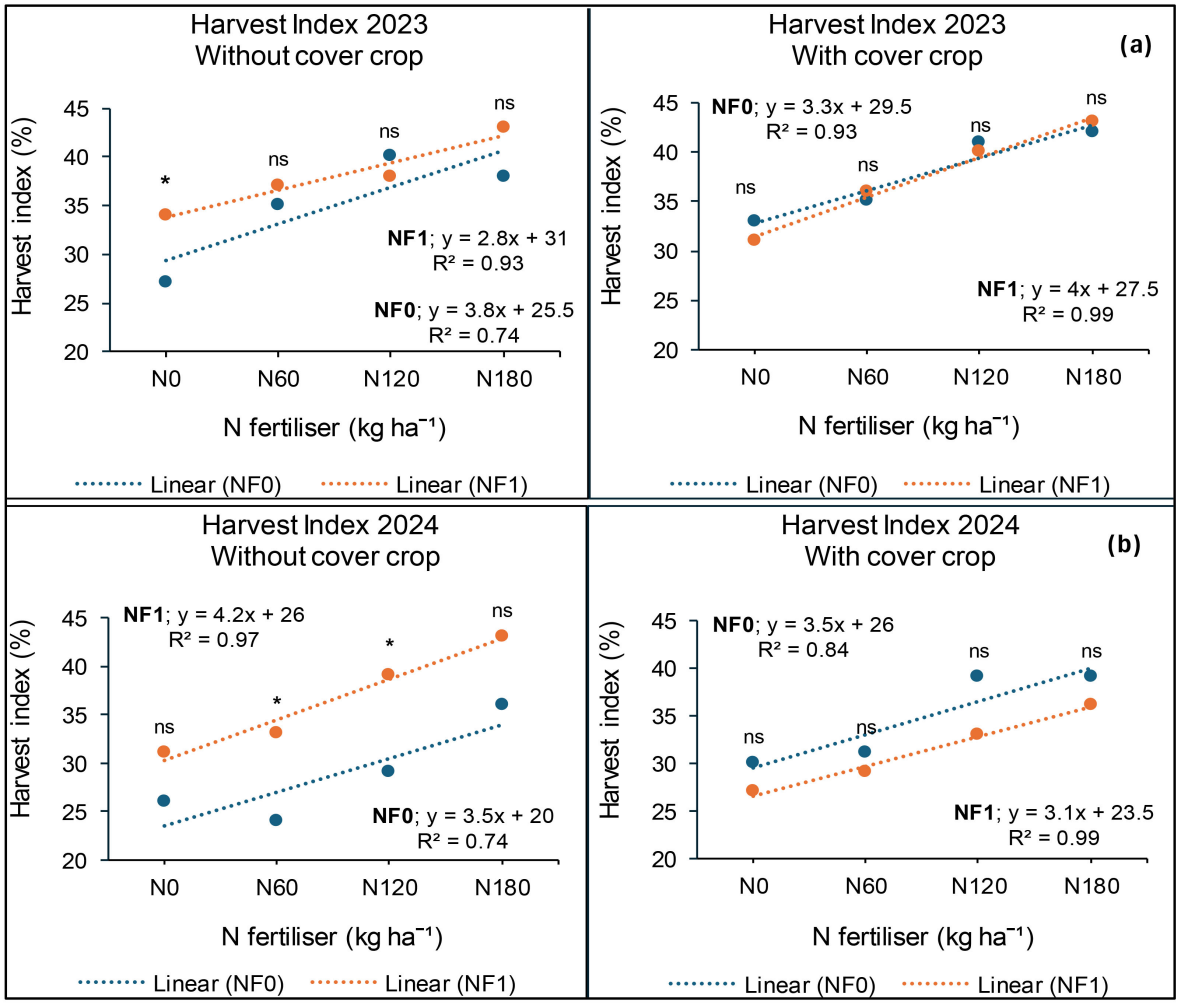
Interactive effects of cover crop, combined nano Zn and Cu and N fertiliser on HI at Syferkuil for the 2023 **(a)** and 2024 **(b)**. NF_0_: without combined nano Zn and Cu; NF_1_: with combined nano Zn and Cu.

The findings at Syferkuil showed that cover crop, combined nano Zn and Cu and N fertiliser, had a significant impact on harvest index (HI) in 2023. Significant interactions were also observed, except for cover crop and N fertiliser. Cover crop incorporation increased HI by 1 percentage point compared with treatments without cover crop (**Figure 17a**). On the other hand, combined nano Zn and Cu improved HI by two percentage points relative to no application (**Figure 17b**). Harvest index at 180 kg N ha^−1^ was two percentage points higher compared with 120 kg N ha^−1^ and 11 percentage points higher than 0 kg N ha^−1^ (**Figure 17c**). Furthermore, without cover crop and combined nano Zn and Cu, N fertiliser at 180 kg N ha^−1^ increased HI by 11 percentage points compared with 0 kg N ha^−1^, but was two percentage points lower than 120 kg N ha^−1^ (**Figure 18b**). However, with combined nano Zn and Cu and no cover crop, HI at 180 kg N ha^−1^ was nine percentage points higher relative to 0 kg N ha^−1^. Incorporating cover crop in the absence of combined nano Zn and Cu resulted in a nine percentage points increase in HI recorded at 180 kg N ha^−1^ compared with 0 kg N ha^−1^ (**Figure 18b**). Meanwhile, the combination of cover crop and nano Zn and Cu increased HI by 12 percentage points at 180 kg N ha^−1^ relative to 0 kg N ha^−1^ (**Figure 18b**). Regardless of cover crop and combined nano Zn and Cu, there was no variation in HI under 180 and 120 kg N ha^−1^.

In 2024, the HI at Syferkuil was significantly affected by combined nano Zn and Cu and N fertiliser, whereas cover crop and all interactions had no impact. Thus, combined nano Zn and Cu increased HI by two percentage points compared with no application (**Figure 17b**). Additionally, HI at 180 kg N ha^−1^ was three percentage points higher than 120 kg N ha^−1^ and nine percentage points greater compared with the average of 0 and 60 kg N ha^−1^ (**Figure 17c**).

### Relationship between grain yield and harvest index at Syferkuil

**Figure 19:**
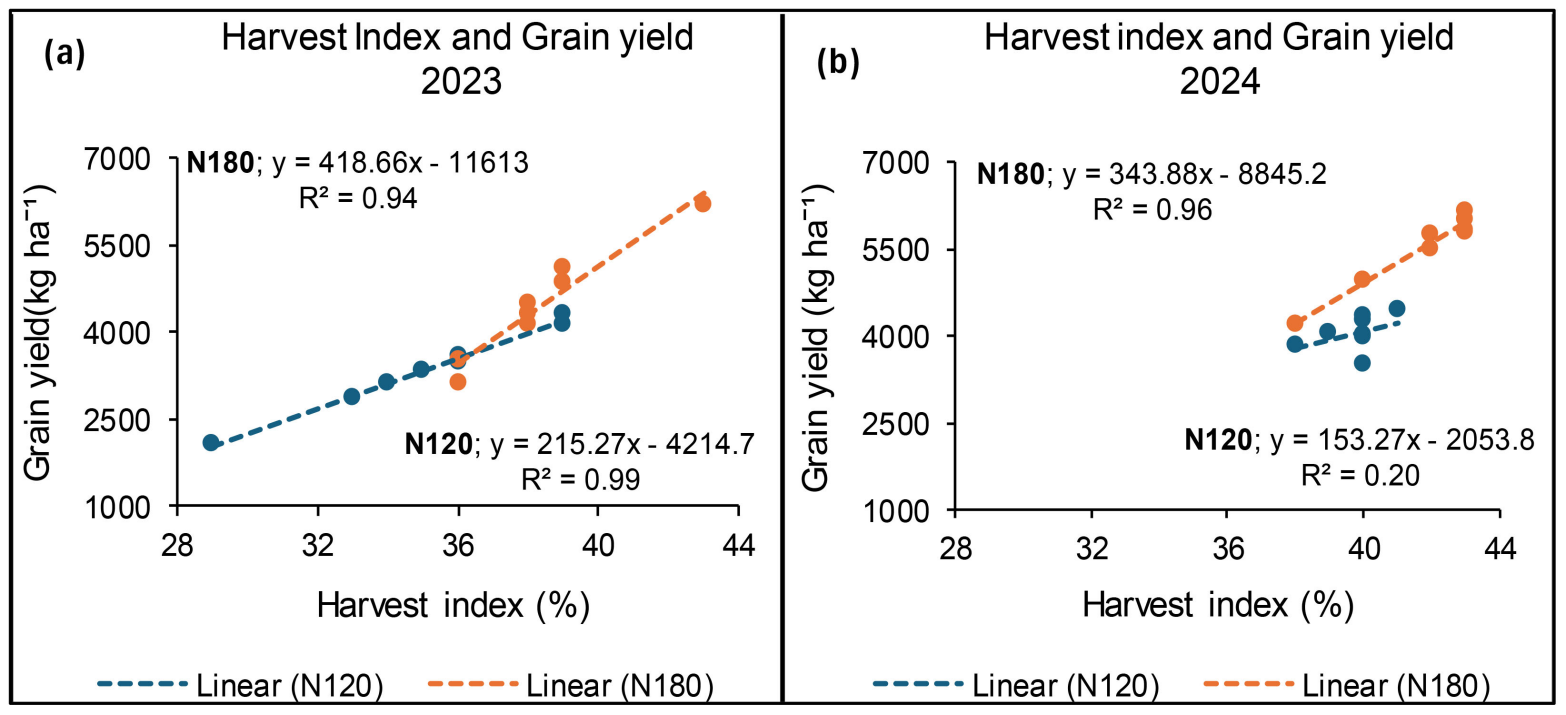
Relationship between grain yield and harvest index for 2023 **(a)** and 2024 **(b)** under N fertiliser application at Syferkuil.

The results for 2023 showed a strong positive relationship between grain yield and HI to the value of 99% and 94% at 120 and 180 kg N ha^−1^, respectively. Similarly, in 2024, a strong relationship between grain yield and HI was recorded at 96% at 180 kg N ha^−1^, whereas 120 kg N ha^−1^ exhibited a weak non-linear relation at 20% (**Figures 19a, b**).

### Harvest index at Ofcolaco

**Figure 20:**
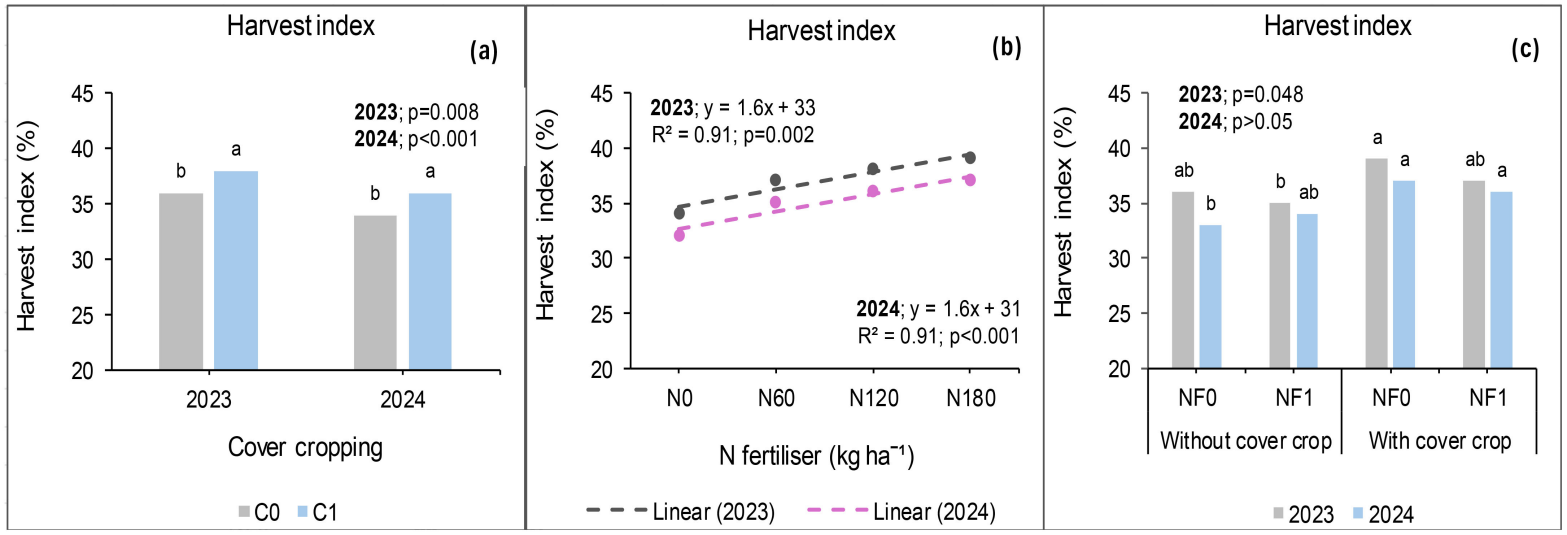
Effects of cover crop **(a)**, N fertiliser **(b)**, and the interaction of cover crop and combined nano Zn and Cu **(c)** on harvest index in 2023 and 2024 at Ofcolaco.

Harvest index at Ofcolaco was significantly affected by cover crop and N fertiliser whereas combined nano Zn and Cu had no impact during the 2023 growing season. Interactive effects were also observed for cover crop and combined nano Zn and Cu whereas others were not significant. Incorporating cover crop improved HI by 2 percentage points compared with no cover crop (**Figure 20a**). Harvest index response to nitrogen fertiliser application of 180 and 120 kg N ha^−1^ were similar, but a 5 percentage points increase was recorded at 180 kg N ha^−1^ compared with 0 kg N ha^−1^ (**Figure 20b**). The interaction effect of cover crop and combined nano Zn and Cu on HI was not significant but variation across treatments ranged from 35% to 37% (**Figure 20c**). In 2024, significant effects of cover crop and N fertiliser were observed on HI at Ofcolaco whereas combined nano Zn and Cu and all interactions were not significant A 12-percentage-point increase in HI was recorded under treatments with cover crop relative to those without it (**Figure 20a**). The harvest index under 180 kg N ha^−1^ application exceeded that of 0 kg N ha^−1^ by five percentage points (**Figure 20b**).

### Relationship between grain yield and harvest index at Ofcolaco

**Figure 21:**
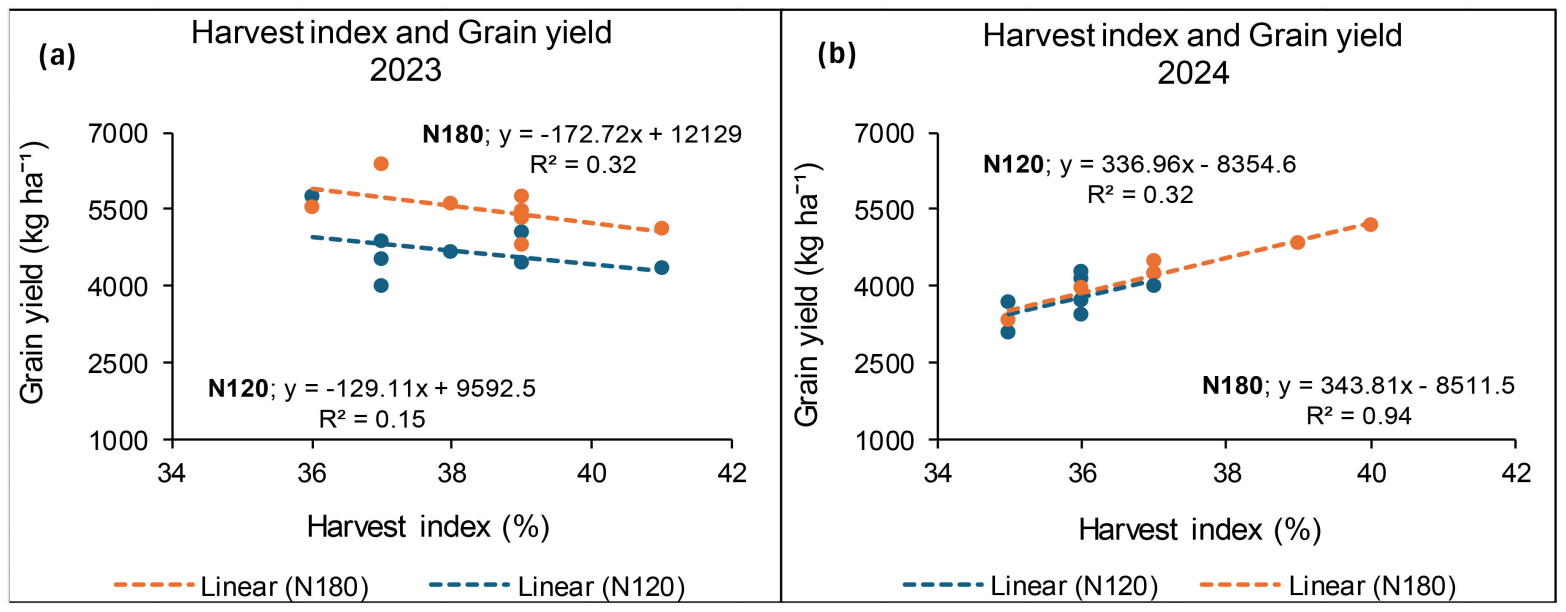
Relationship between grain yield and harvest index for 2023 **(a)** and 2024 **(b)** at Ofcolaco.

In 2023, a weak linear relationship between grain yield and HI was recorded at 32% and 15% for 180 and 120 kg N ha^−1^, respectively. Meanwhile, in 2024, a strong positive relationship (94%) between HI and grain yield was recorded at 180 kg N ha^−1^. However, 120 kg N ha^−1^ had a weak positive relation of 32% (**Figures 21a, b**).

### Principal component analysis for selected canola yield parameters at Syferkuil

**Figure 22:**
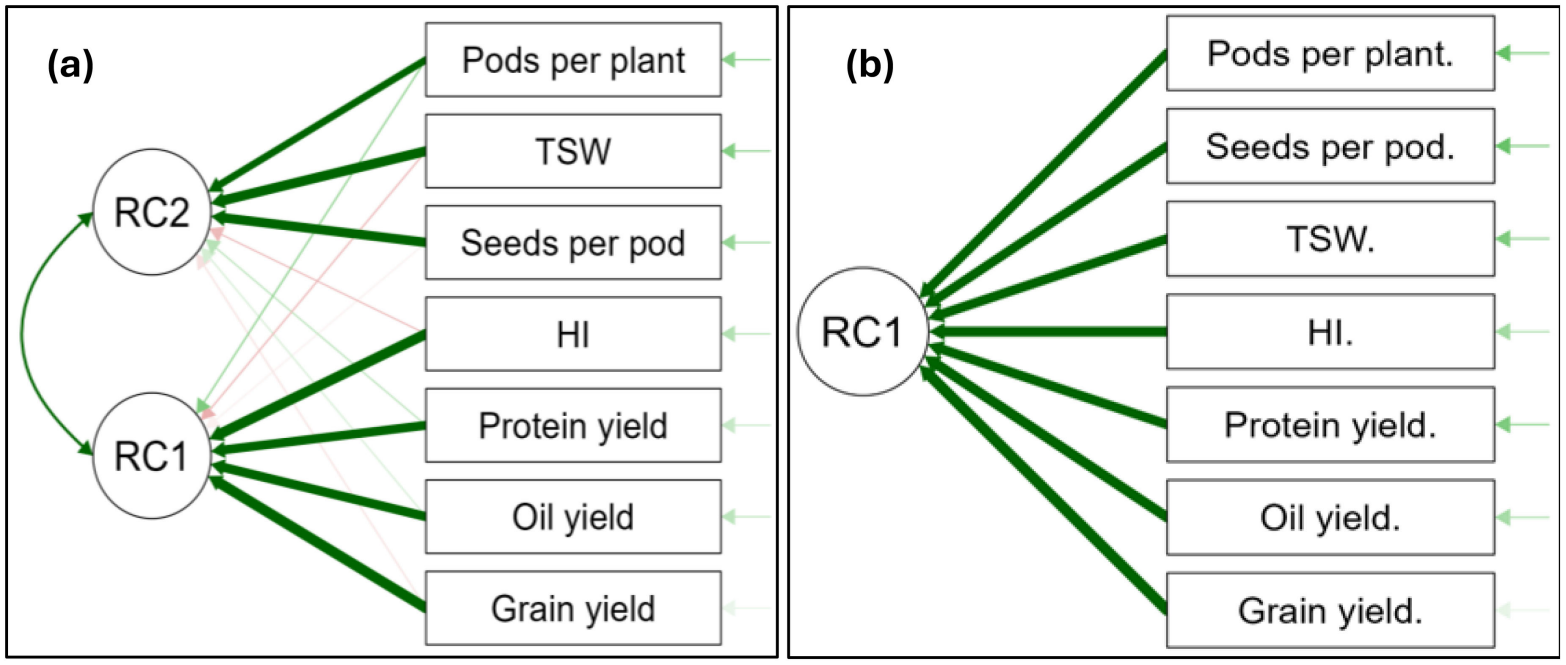
Principal component analysis of significant factors of selected yield parameters at Syferkuil for 2023 **(a)** and 2024 **(b)**. Thick green arrows: strong positive relationship; thin green arrows: weak positive relationship; Thick red arrows: strong negative relationship; thin red arrows: weak negative relationship.

During the 2023 season, the principal component analysis revealed two rotated components (RC1 and RC2) which accounted for 87.4% of the total variance in the data (**Figure 22a**). In RC1, grain yield, HI, oil yield, and protein yield had strongly significant positive loadings. Meanwhile, TSW, number of seeds per pod, and number of pods per plant also indicated strong positive loadings with RC2. Rotated components 1 and 2 each, respectively, explained 67.1% and 20.3% of variation in the parameters. In 2024, only one rotated component (RC1) was noted, which explained 83% of the total variance in all variables combined (**Figure 22b**). This component shows strong positive loadings of the grain yield, oil yield, protein yield, TSW, number of seeds per pod, and number of pods per plant. The strong positive loadings explain a substantial portion of the variables, further showing a synergistic relationship with the RC’s.

### Principal component analysis for selected canola yield parameters at Ofcolaco

**Figure 23:**
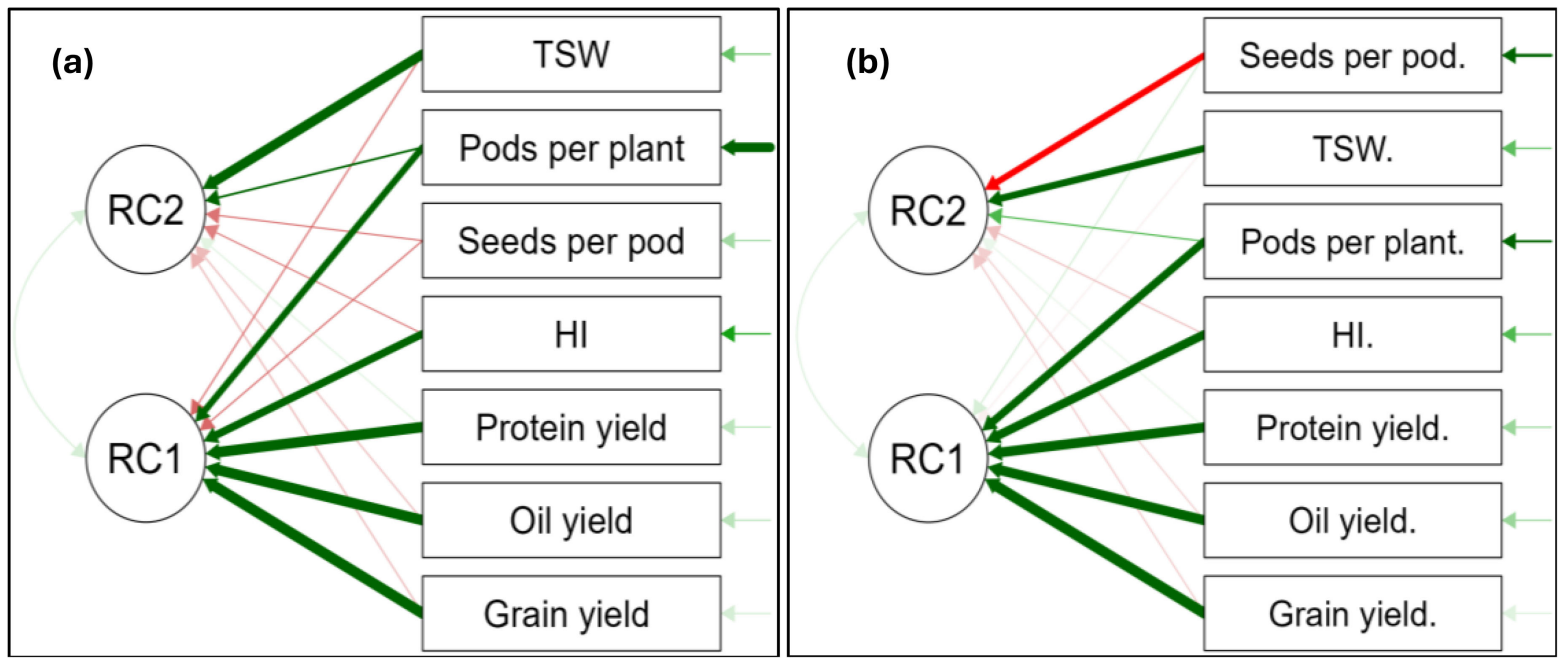
Principal component analysis of significant factors of selected yield parameters at Ofcolaco for 2023 **(a)** and 2024 **(b)**. Thick green arrows: strong positive relationship; thin green arrows: weak positive relationship; Thick red arrows: strong negative relationship; thin red arrows: weak negative relationship.

In 2023, two rotated components RC1 and RC2 were identified and collectively accounted for 72.6% of the variance (**Figure 23a**). Grain yield, oil yield, protein yield, HI, and number of pods per plant showed strong positive loadings under RC1, which explained 56.5% of their variation. Conversely, TSW and number of pods per plant were positively loaded under RC2, explaining 16.1% of their variance. However, the number of seeds per pod was not explained by either RC1 or RC2 due to very high uniqueness (89%). During the 2024 season, rotated components 1 and 2 collectively accounted for 73.7% of the total variance (**Figure 23b**). The rotated component (RC1) was found to explain 57.3% of the variance in grain yield, oil yield, protein yield, HI, and number of pods per plant showing strong positive loadings. Thousand seed weight (TSW) also loaded positively for RC2, which explained 16.4% of variation. A very strong negative loading was observed for number of seeds per pod under RC2.

## Discussion

### Grain yield

The research showed that sunn hemp cover crop significantly increased grain yields except at Syferkuil in 2024, aligning with Khemtong et al. (2023) reporting a 1,167-kg ha^−1^ Japonica rice yield using the same cover crop. Dou et al. (2016) indicated that location variance can reveal contrasting effects of cover crops on soil fertility, which affected yields in 2024. The overall favourable response to the incorporation of sunn hemp as a cover crop presents a possibility for its widespread application in cropping systems. The leguminous cover crop, possessing nitrogen-fixing abilities, could significantly benefit farming systems in Limpopo province, particularly for smallholder farmers, where poor soil fertility and land degradation pose serious constraints to sustainable crop production. On the other hand, nano Zn and Cu application enhanced grain yield by 15% and 17% at Syferkuil in 2023 and 2024, respectively, consistent with findings from Sohail et al. (2022) and Tarafdar et al. (2014) regarding positive responses of canola. This positive response, however, has not been found in a crop such as wheat (Kopittke et al., 2019). Nitrogen fertiliser at 120 kg N ha^−1^ significantly boosted canola yield, accounting for 70% to 85% of the maximum application of 180 kg N ha^−1^, yielding 3,000 to 4,500 kg ha^−1^, surpassing the global average of 2.7 to 3.4 t ha^−1^ (Lin et al., 2020; Du Toit, 2018). The study highlights the potential for canola production in Limpopo province, where it is currently limited to the Western Cape (DAFF (Department of Agriculture, Forestry and Fisheries, South Africa), 2019; Galal, 2021). The yield drop in 2024 was likely due to lower rainfall and higher temperatures affecting crop growth (Lin et al., 2020; Khan et al., 2017). Nano Zn and Cu combined with N fertiliser also improved yields at Ofcolaco, corroborating Upadhyay et al. (2023), yet current research lacks extensive field trials on nanofertilisers for canola. Our field studies may facilitate further research to leverage the positive effects of nano-micronutrient application in crop production.

### Oil yield

Canola oil yield showed a significant positive response to sunn hemp cover crop incorporation, except at Syferkuil in 2024. This effect of cover crop in this study is supported by Sitthaphanit et al. (2009), who recommended the incorporation of cover crop in canola production to boost soil fertility. The impact of sunn hemp cover crop was anticipated, considering the benefits associated with leguminous crop residues in agricultural practices (Awelewa et al., 2024). Kopittke et al. (2019) reported that nanofertilisers sometimes lacked advantages over conventional fertilisers, which was evident in this study concerning oil yield and several other parameters across locations and seasons. Oil yield in this study significantly varied with different N fertiliser rates at both sites, with a slight decline at the highest rate. The study found that the highest oil yield was achieved at 120 kg N ha^−1^ application rate, which was comparable with 180 kg N ha^−1^ at Syferkuil, suggesting 120 kg N ha^−1^ as optimal for canola oil production. Furthermore, the study revealed that applying 180 kg N ha^−1^ was excessive and decreased oil yield; however, reducing the application to 120 kg N ha^−1^ can maintain oil yield without compromising the environment. Similar findings of decreased oil yields due to protein build-up at high application rates have been reported (Gomaa and Fayed, 2024; Yahbi et al., 2022). The decline in oil yield at 180 kg N ha^−1^ in this study is also corroborated by Zhuo et al. (2024), who stated that although higher application rates can increase seed yield, the oil yield is usually compromised. The correlation between canola grain yield production and oil concentration is intricate, sometimes necessitating a trade-off. For instance, elements that enhance one may diminish the other, but methods exist to augment both simultaneously. Achieving both through breeding programmes could be beneficial to farmers in the Limpopo province.

### Protein yield

During the 2023 and 2024 growing seasons, cover crop incorporation generally increased protein yield at both study sites, excluding Syferkuil in 2024. Dou et al. (2016) noted varied effects of cover crop on soil fertility which is dependent on location and use duration. Given the limited research conducted on nano Zn and Cu, their overall effect in this study could be due to their nanoscale properties enhancing root exudation of essential organic acids (Faizan et al., 2021). Syferkuil demonstrated a notable increase in protein yield with combined nano Zn and Cu, achieving a 16% and 27% increase for 2023 and 2024, respectively. Similarly, Sohail et al. (2022) and Tarafdar et al. (2014) reported positive effects of Zn nanofertiliser on plant growth, yield and their components. In contrast, Ofcolaco did not show any impact of nano Zn and Cu on protein yield, consistent with Kopittke et al. (2019) who reported no improvements in growth and yield parameters following the application of nanofertiliser. As such, Ndaba et al. (2022) highlighted that various factors affect nanofertiliser use efficiency. Additionally, increased protein yield was associated with higher N fertiliser application rates during both seasons, with 180 kg N ha^−1^ yielding the highest results. Gomaa and Fayed (2024) supported that higher N fertiliser application improves protein yields and amino acid composition. The combined effects of cover crops, nano Zn and Cu, and N fertiliser also bolstered protein yield at Syferkuil in 2024, although literature on this combination is lacking.

### Yield components

#### Number of pods per plant

The impact of cover crops, particularly leguminous species such as sunn hemp, enhances soil fertility, benefiting crop growth and yield components (Sitthaphanit et al., 2009). However, in this study, a positive response of the number of pods per plant to cover crop incorporation was only observed at Ofcolaco during the 2024 growing season. Notably, this effect was observed solely in one season at Ofcolaco, suggesting that seasonal and climatic variations between the study sites could have influenced the results. Additionally, combined nano Zn and Cu positively affected the number of pods per plant, likely due to the critical role of Zn in chlorophyll synthesis and electron transport, which ultimately enhances plant growth (Sohail et al., 2022). Faizan et al. (2021) support the use of Zn-based nanofertilisers, indicating improved plant growth relative to conventional bulk fertilisers. Although the application of combined nano Zn and Cu micronutrient in canola production is a first of its kind, several studies have reported on the overall positive effect of nanofertilisers on plant growth (Tarafdar et al., 2014; Raliya et al., 2015; Meena et al., 2017). Nitrogen fertiliser application increased the number of pods per plant at both study sites during the 2023 and 2024 seasons. The highest count was at 180 kg N ha^−1^, consistent with Li et al. (2019), while Gomaa and Fayed (2024) noted more at 120 kg N ha^−1^. Sible et al. (2021) linked higher N fertilisation to increased vegetative growth in canola, ultimately enhancing the number of pods per plant and several yield components.

#### Thousand seed weight

Thousand seed weight was improved with the application of combined nano Zn, Cu, and N fertiliser at Syferkuil. This finding is corroborated by Jaksomsak et al. (2017), who demonstrated that the presence of the Zn micronutrient is advantageous for seed growth and development. Furthermore, this study indicates that higher nitrogen rates significantly affect TSW compared with lower rates. This observation is consistent with the findings of Yahbi et al. (2022), who reported a 0.6-g increase in TSW in canola at 90 kg N ha^−1^ relative to 0 kg N ha^−1^. However, TSW at Ofcolaco was not influenced by any of the treatment factors. The sole application of sunn hemp cover crop did not affect TSW, but its interaction with N fertiliser had a positive outcome, which could have resulted from the effect of nitrogen on the parameter. Khan et al. (2017) stated that the observed increase in some yield components, such as TSW, is attributed to higher N fertilisation rates, a trend that was observed in this study. The positive response to combined nano Zn and Cu interaction with N fertiliser could be due to their collective effect arising from the synergy of the nutrients in boosting plant metabolic function.

#### Number of seeds per pod

The study found that sunn hemp cover crop and N fertiliser positively affected the number of seeds per pod, varying across location and seasons. The increase in seed per pod under cover crop incorporation could have resulted from the enhanced microbial activity, which improves the workability of the soil through organic matter addition, supporting plant growth (Gong et al., 2021). The minimal impact of cover crops on the number of seeds per pod at Syferkuil may result from insufficient organic matter accumulation or the prevailing climatic and soil conditions. Treatments with higher N fertiliser were more influential on the number of seeds per pod compared with those with lower rates, an outcome which was also observed by Kamkar et al. (2011) who reported a 25% increase from 0 to 270 kg N ha^−1^. Yahbi et al. (2022) also observed a higher number of seeds per pod at higher N fertiliser rates, indicating that the improvement may be due to increased leaf growth, which plays a huge role in pod and seed development. However, at Ofcolaco, the number of seeds per pod did not respond to N fertilisation, an observation supported by Yahbi et al. (2022), who stated that sometimes the number of seeds per pod does not respond to N fertiliser regardless of the application rates.

### Relationships between grain yield and the measured parameters

The strong relationship observed between grain yield and the number of pods per plant, seeds per pod, and TSW was expected given the contribution of all the parameters towards grain yield. However, these associations showed great variation in response to N fertiliser application at each location and the seasons. The application of 120 and 180 kg N ha^−1^ of fertiliser demonstrated comparable effects on these relationships, although in certain instances, one rate outperformed the other. The strong relationships observed between grain yield and some of the yield components were expected, given the contribution of N fertiliser towards achieving optimal grain yield outputs (Eibad et al., 2022). However, the similarities between 120 and 180 kg N ha^−1^ regarding the relationships further indicate the potential of obtaining optimum grain yield with reduced fertiliser application rate. The difference in relationships among the parameters, which is specific to each location, could also be explained by the fact that 27% of winter canola yield variations are caused by genetics or its interaction with the environment (Assefa et al., 2014).

### Harvest index (%)

Harvest index is a key physiological parameter indicating vegetative growth efficiency impacting economic yield. In both seasons and locations, this study confirmed that HI was significantly affected by cover crop, combined nano Zn and Cu and N fertiliser application, although the effects varied by location. The harvest index ranged from 33% to 38% with cover crop, 34% to 38% with nano Zn and Cu, and from 29% to 42% with N fertiliser across seasons and locations, showcasing improvement over findings by Gomaa and Fayed (2024), which ranged from 25% to 31% under N fertilisation. Increased HI linked to sunn hemp cover crop is likely due to nutrient release fostering optimal growth conditions for the canola (Koudahe et al., 2022). The trend of increasing yield with elevated N fertiliser rates also corresponded with HI increases, emphasising a linear relationship between HI and grain yield, primarily linked to the role of nitrogen in canola growth (Kamkar et al., 2011). Enhanced biomass production and nutrient uptake positively influenced HI, aligning with previous studies (Raman et al., 2019; Godebo et al., 2021). The increased HI at higher fertiliser rates observed in this study agrees with the findings of Rafiqul et al. (2018), who also noted higher HI under 180 kg N ha^−1^. While literature on nano Zn and Cu’s influence is limited, positive results were recorded, especially with combined treatments, reflecting beneficial properties of nanofertilisers (Yassen et al., 2017). Considering the high N fertiliser requirements of canola, which is about 25% more than that of wheat (Yahbi et al., 2022), the overall HI observed in this study was reasonable.

## Conclusions and recommendations

This study was established to examine the potential of sunn hemp cover crop and the combined application of nano Zn and Cu in reducing nitrogen fertiliser application in canola production. The results indicated a favourable response of grain yield, oil yield, protein yield, harvest index, number of pods per plant, and seeds per pod to sunn hemp cover crop. Additionally, the combined application of nano Zn and Cu with nitrogen fertiliser enhanced grain and protein yield, harvest index, TSW, and number of pods per plant, with variations across seasons and locations. The study further revealed that the highest fertiliser rate of 180 kg N ha^−1^ increased grain yield and yield components. However, the response of these parameters at 120 kg N ha^−1^ was similar to that of 180 kg N ha^−1^, suggesting the potential to downsize the N fertiliser input. The results further suggest that the application of 120 kg N ha^−1^ is ideal for canola production, given its influence on grain and oil yield, and some yield components. Contrarily, the highest oil yield was found at application rates of 120 and 180 kg N ha^−1^. Future research should extensively evaluate canola production following continuous cover crop incorporation and varied doses of nano Zn and Cu at lower N fertiliser rates. Soil health indicators should also be monitored to establish the residual effects of the treatments on soil properties and how it impacts future management practices relating to canola.

## Data Availability

The original contributions presented in the study are included in the article/**Supplementary Material**. Further inquiries can be directed to the corresponding author.
