# Supplementary figures and images for: Canola grain yield and quality response to Sunn hemp cover crop, combined nano zinc and copper, and nitrogen fertiliser application under different agroecological zones
Source: Front Plant Sci. 2025 Dec 1;16:1706625. doi: 10.3389/fpls.2025.1706625 (PMC12703711; doi:10.3389/fpls.2025.1706625)

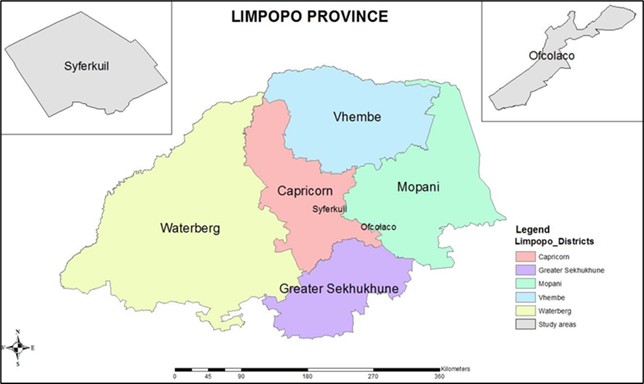

Supplement: Supplementary file 1 [file Image1.jpeg]

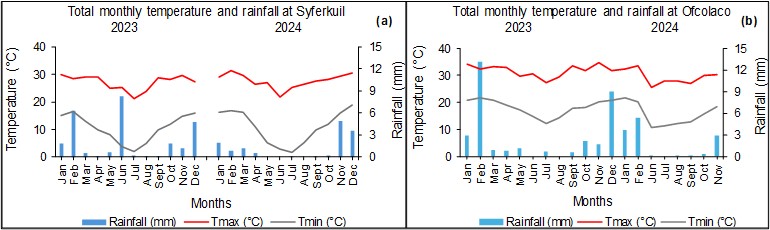

Supplement: Supplementary file 2 [file Image2.jpeg]
